# Phylogeography of the termite *Macrotermes gilvus* and insight into ancient dispersal corridors in Pleistocene Southeast Asia

**DOI:** 10.1371/journal.pone.0186690

**Published:** 2017-11-29

**Authors:** G. Veera Singham, Ahmad Sofiman Othman, Chow-Yang Lee

**Affiliations:** 1 Urban Entomology Laboratory, Vector Control Research Unit, School of Biological Sciences, Universiti Sains Malaysia, Minden, Penang, Malaysia; 2 Population Genetics Laboratory, School of Biological Sciences, Universiti Sains Malaysia, Minden, Penang, Malaysia; National Cheng Kung University, TAIWAN

## Abstract

Dispersal of soil-dwelling organisms via the repeatedly exposed Sunda shelf through much of the Pleistocene in Southeast Asia has not been studied extensively, especially for invertebrates. Here we investigated the phylogeography of an endemic termite species, *Macrotermes gilvus* (Hagen), to elucidate the spatiotemporal dynamics of dispersal routes of terrestrial fauna in Pleistocene Southeast Asia. We sampled 213 termite colonies from 66 localities throughout the region. Independently inherited microsatellites and mtDNA markers were used to infer the phylogeographic framework of *M*. *gilvus*. Discrete phylogeographic analysis and molecular dating based on fossil calibration were used to infer the dynamics of *M*. *gilvus* dispersal in time and space across Southeast Asia. We found that the termite dispersal events were consistently dated within the Pleistocene time frame. The dispersal pattern was multidirectional, radiating eastwards and southwards out of Indochina, which was identified as the origin for dispersal events. We found no direct dispersal events between Sumatra and Borneo despite the presence of a terrestrial connection between them during the Pleistocene. Instead, central Java served as an important link allowing termite colonies to be established in Borneo and Sumatra. Our findings support the hypothesis of a north-south dispersal corridor in Southeast Asia and suggest the presence of alternative dispersal routes across Sundaland during the Pleistocene. For the first time, we also propose that a west-east dispersal through over-water rafting likely occurred across the Pleistocene South China Sea. We found at least two independent entry routes for terrestrial species to infiltrate Sumatra and Borneo at different times.

## Introduction

Southeast Asia (SEA) harbours 20–25% of the planet’s plant and animal species and is thus a highly biodiverse region [1–2]. SEA today is relatively small and fragmented compared to the past few million years. During the Pliocene–Pleistocene glacial periods, the land area was almost twice as large, as the mean sea level was 62 m below today’s level. At that time, the now submerged Sunda shelf formed a continuous landmass from which islands formed as the sea level rose to connect the islands and mainland of SEA into a large continental block known as Sundaland [3–5]. This continuous landmass is thought to have enabled migration of many organisms across the Sunda region (e.g., [6–7] for mammals; and [8] for snakes and frogs). The dispersal and distribution of Sundaland’s terrestrial fauna likely depended on the type and extent of vegetation and soil structure on this landmass. Much evidence suggests that the lowlands of SEA were dominated by open vegetation landscapes, including savanna, grassland, and open woodland, through much of the Pleistocene glacial period [9–14], although conflicting arguments that a large continuous rainforest instead of savanna was present even under full glacial conditions were also reported [15–17], and this feature is still intensely debated [18]. The presence of multiple early and middle Pleistocene archaeological sites containing open vegetation-adapted hominins and other mammals in Java [19–21] suggests that early faunal migration required crossing and surviving the open vegetation landscapes on mainland SEA [21].

**Fig 1:**
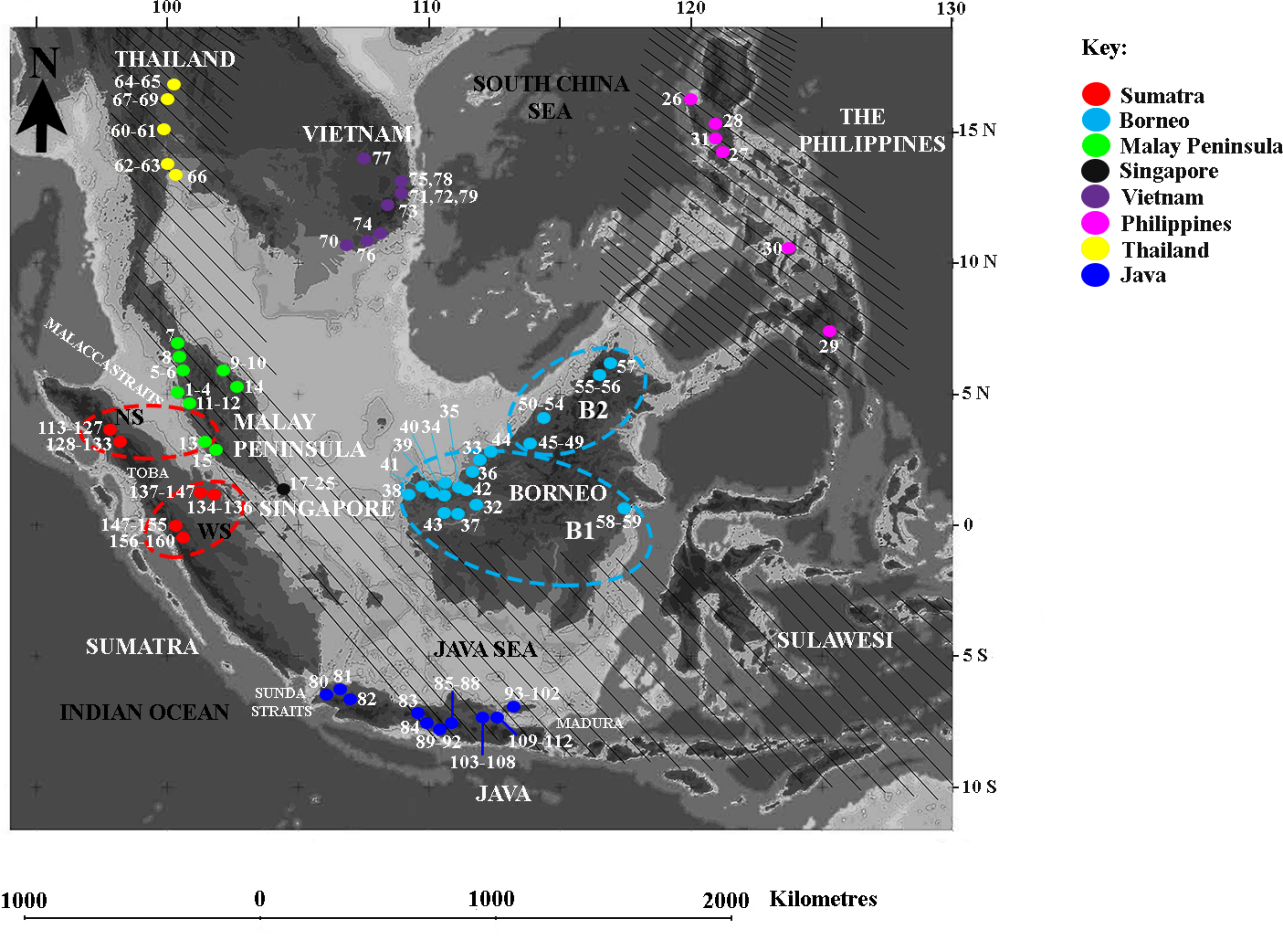
Southeast Asia showing the distribution of *M*. *gilvus* colonies used in mtDNA analysis sampled from 66 localities. Labelled numbers represent individual colony numbers as listed in S1 and S2 Tables. The maximal extent of Sundaland during the last glacial maximum is shown in light gray. Areas covered by the diagonal lines represent the savanna corridor proposed by Heaney [9]. The broken lines represent phylogeographic subdivisions based on mtDNA analysis in Borneo (blue) and Sumatra (red) from this study (see text). The base map was reprinted from Sathiamurthy & Voris [22] under a CC BY licence, with permission from Dr. Harold K. Voris, 2006 Field museum of Natural History, Chicago, Illinois, USA.

Several hypotheses regarding the size of open vegetation dispersal corridors in Sundaland have been proposed. Heaney [9] first postulated a north-south savanna corridor extending down the Malay Peninsula and across the now marine region between Borneo and Java (Fig 1). In this scenario, Palawan and the Philippines are also considered to have been largely covered by savanna (Fig 1). Subsequently, two alternative interpretations of savanna corridors were proposed in a review by Bird et al [11]: a narrow but continuous 50–150 km wide band of open vegetation running along the sand-covered divide between the present day South China and Java Seas through the Malacca Straits and a broader corridor expanding from the eastern slope of the Sumatran highlands well into the interior of western Borneo. On contrary, Slik et al. [16] found no evidence for a savanna corridor between Borneo and Sumatra, but show a coarse soil barrier in the then-exposed sea floor. Identifying such corridors and understanding their role as dispersal routes is therefore important for understanding the causes and development of modern biogeographical patterns in the region, including early humans’ ability to exploit such corridors for dispersal.

Here we reconstruct the potential dispersal corridors for an extant subterranean fungus-growing termite species, *Macrotermes gilvus* (Hagen). Termites are ideal subjects for biogeography and evolutionary studies because they are easily sampled, feed and live in a broad range of landscapes and are sensitive to environmental disturbances. Furthermore, colony maturation is slow and dispersal of reproductive alates is short ranged [10]. *M*. *gilvus* was particularly suitable for this study for several reasons: 1) it has a vast distribution in the lowlands of tropical SEA and can be found as far west as India [23]. Recent findings by Bourguignon et al. [24] show that Asian *Macrotermes* arose around 20 Ma. The location and timing of divergence of this genus in SEA is well suited to investigate the effect of past geological events in shaping the distribution of the local taxa; 2) dispersal flight range is short and establishment of new colonies depends primarily on soil structure and the availability of the obligate mutualistic fungal symbiont, *Termitomyces* sp.. Therefore, the genetic signature of this species should reflect historical patterns rather than contemporary gene flow between different regions of SEA; and 3) *M*. *gilvus* can inhabit both lowland forest habitat and the soil in open vegetation environments or urban settings, such as parks, underneath trees with an open-canopy along roadsides, in areas where vegetation is dominated by grasses, or in agricultural sites such as paddy field terraces and oil palm plantations formerly covered by rainforest [25–29]. Therefore, a wide range of vegetated Pleistocene land connection is suitable for *M*. *gilvus* dispersal, making it a suitable candidate for defining the extent of early dispersal corridors in pre-historic SEA. In this study, we used both nuclear and mitochondrial DNA markers to infer the evolutionary history of, and dispersal pathways, for the species. The genetic patterns observed allow inferences to be drawn regarding the palaeoenvironmental history and spatiotemporal dynamics of ancient dispersal routes of the region.

## Materials and methods

### Sampling strategy

We used two independently inherited molecular markers (mtDNA and microsatellites) to accurately reflect population history. Two hundred and thirteen *M*. *gilvus* colonies were sampled in the field from 66 locations around SEA of various habitat types mainly from the urban environment and agricultural sites (see Fig 1, S1 and S2 Tables). Samples from 160 colonies were used for mtDNA analysis and all 213 colonies were used for microsatellite analysis (S1 and S2 Tables). *M*. *gilvus* colonies are founded by a pair of mating reproductives, and therefore we regarded each colony as a single sampling unit. Only one individual per colony was used for mtDNA and microsatellite analyses. The field studies did not involve endangered or protected species and no specific permissions were required for field studies done in public parks and roadsides. For agricultural sites, the owner of the land has given permission to collect samples for the study.

### Mitochondrial DNA analysis

Worker termites were pulverized in liquid nitrogen, and genomic DNA was extracted following the CTB Tissue Extraction Kit protocol (Intron, Gyeonggi-do, Korea). Each DNA sample was polymerase chain reaction (PCR) amplified at two mitochondrial DNA regions: the partial cytochrome c oxidase subunit II (COII) and the large ribosomal subunit RNA (16S rRNA) genes. The PCR conditions and primer information can be found in S1 Appendix.

Phylogenetic relationships among mtDNA haplotypes were constructed using three different approaches: maximum parsimony (MP), maximum likelihood (ML), and Bayesian inference (BI). MP analysis was performed using the tree-bisection-reconnection branch-swapping algorithm and 1,000 random taxon addition replicates under a heuristic search in paup* [30], saving no more than 100 equally parsimonious trees per replicate. Nonparametric bootstrap values for branch support were assessed using 100 bootstrap pseudo-replicates with 10 random taxon additions in each replicate. ML analyses were conducted in garli 2.0 [31] with two runs of 100 replicates; each was executed in the best-fit substitution model as determined in Modeltest 3.7 [32] (S3 Table) and incorporated the best partitioning scheme selected based on Akaike’s information criterion using different subset rates and 5,000 nonparametric bootstrap iterations (S4 Table). To mitigate any possible effect of over-parameterization in data partitions, an alternative ML analysis was performed in raxmlGUI 1.3 [33], which incorporated a more generalized GTR + G substitution model with rate heterogeneity across all data partitions using the “ML + thorough bootstrap” option with 10,000 bootstrap replicates under the rapid hill-climbing algorithm. Similarly, BI was conducted in MrBayes 3.1 [34] and incorporated the best partitioning scheme based on the bayes factor analysis (S5 Table).

Two independent runs starting from different random trees were performed using partition-specific rates, and four MCMC chains were run for 25 million generations with sampling frequency every 1000 generations (burn-in = 6250). Convergence was assessed by observing when the standard deviation of split frequencies fell below 0.01. Gaps were treated as missing data. Non-fungus-growing termites’ mtDNA sequences from Genbank (*Drepanotermes sp*. (JX144938.1), *Macrognathotermes errator* (JX144939.1), and *Nasutitermes triodiae* (JX144940.1)) were used as outgroups in all phylogenetic trees.

Alternative phylogenetic hypotheses for evaluating the monophyly of clades in the phylogenetic tree using constrained tree topologies were tested by calculating the probabilities of tree topologies using the approximately unbiased (AU), Kisino-Hasegawa (KH), weighted KH (WKH), Shimoidara-Hasegawa (SH), and weighted SH (WSH) tests that were determined from the multi-scale bootstrap in consel [35]. A *P*-value of < 0.05 was interpreted as strong evidence for rejecting the phylogenetic hypothesis.

A statistical parsimony network was reconstructed using the software tcs 1.21 [36], which links haplotypes through a series of evolutionary steps based on algorithms that estimate the 95% reconnection limit between haplotypes. This topology was compared with a minimum spanning network produced by the median-joining method in Network 4.6.1.1 [37]. We made such comparisons so that any conflicting results could be identified and subsequently interpreted with caution. Additionally, pairwise *Φ*_*ST*_ between pairs of populations was compared in Arlequin 2.0 [38] using 10,000 permutations at α = 0.05.

Phylogeographic reconstruction of the spread of *M*. *gilvus* through space and time was conducted using the Bayesian MCMC approach in the beast 2.0 software package [39] that includes a discretized spatial diffusion model that can quantify the rate at which termite lineages moved between sampled locations [40]. Analyses were carried out using an uncorrelated lognormal relaxed molecular clock model that allows rates to vary among branches. Three sets of analyses were conducted using an insect mitochondrial substitution rate with an upper limit of 2.28% Myr^–1^ and a lower limit of 0.9% Myr^–1^ as well as a standard invertebrate mitochondrial divergence rate of 1.5% Myr^–1^ as the rough median; a similar approach according to Schutze et al [41]. Two runs of 30 million generations, each incorporating the GTR + G nucleotide substitution model, were run for each analysis. The resulting geotree files were combined and annotated with location states in TreeAnnotator 1.6.1 [39] before being converted into a keyhole markup language (KML) file using spread [42] and subsequently visualized as animation in the free web service Google Earth [43] The software Tracer 1.6 [44] was used to monitor support for stationary and efficient mixing, as diagnosed using effective sample size statistics. The discrete phylogeographic analysis required that each sequence be assigned a specific character state based on its geographic origin. In this study, we considered movement among major countries and regions (15 character states: Thailand, Vietnam, the Philippines, the Malay Peninsula, Singapore, North Sumatra, Riau, West Sumatra, West Java, Central Java, East Java, Madura, Southwest Borneo (B1), Northwest Borneo (B2), and East Kalimantan).Where more than two locations were grouped, the latitude and longitude of the centroid of the polygon defined by them was used.

Divergence dating using fossil calibrations from an inter-species termite phylogeny was conducted separately to attain an alternative resolution of divergence times. A set of 101 termite taxa containing the ~681 bp COII gene haplotypes derived from this study as well as additional sequences obtained from previous studies [45–46] aimed at dating the related fungus-growing termite group (subfamily Macrotermitinae) was used to construct a starting tree in the software MrBayes 3.1 [34] using GTR + G nucleotide substitution models. Only the COII gene was used due to lack of complementary sequences from the 16S rRNA gene for other macrotermitines. Divergence time was estimated using the Bayesian relaxed-clock uncorrelated exponential approach as implemented in beast 1.7.4 [39] based on fossil calibration procedures from previous published works [45–46] (S1 Fig).

### Microsatellite analysis

Multiplex PCR was conducted using 15 novel polymorphic microsatellite loci developed previously for *M*. *gilvus* following PCR conditions and fragment analysis as detailed in Veera Singham et al [47]. Phylogenetic inference of microsatellite genotypes was initially deduced without *a priori* assumptions of population definition based on the genetic distance measures (Dps’ and Dkf’) generated with the [1-ps/kf] option in microsat [48] that were used to construct a neighbor-joining (NJ) phylogenetic tree with the program neighbor in the phylip 3.69 package [49]. Additionally, another NJ tree was reconstructed to view relationships between pre-defined populations groups as inferred from microsat [48] using *D*_*A*_ distance in poptree2 [50] at10,000 bootstrap iterations.

The probability of individual assignments to population clusters (*K*) without prior information about the origin of individuals was calculated using structure 2.3 [51] under the assumption of an admixture model and correlated allele frequency with the lambda parameter set to one, as the user’s manual advises; the length of burn-in for MCMC chains was set to 10,000 each. We performed 20 runs for each dataset to determine the amount of variation of the likelihood for each *K* ranging from one to 15, and the most probable number of groups was determined as described in Evanno et al [52]. distruct [53] was used for producing the plot for visualization.

Multiple measures of genetic diversity (expected heterozygosity (*H*_E_), observed heterozygosity (*H*_O_), average number of alleles per locus and size range per locus, departures from Hardy–Weinberg equilibrium (HWE), and genotypic linkage disequilibrium) were analysed using Arlequin [38]. Detection of null alleles and scoring bias due to stuttering or large allelic dropouts was performed using Micro-Checker 2.2.3 [54]. The inbreeding coefficient (*F*_*IS*_) and allelic richness (A_R_) as a standardized measure of the number of alleles per locus independent of the sample size based on rarefaction method were assessed using fstat 2.9.3. [55]. Assessments of population pairwise comparisons using *F*_*ST*_ and sum of squared size differences (*R*_*ST*_) were derived from Arlequin [38]. Isolation-by-distance analysis was conducted for both mtDNA and microsatellite analyses by regressing the Euclidean distance between the geographic localities and the population’s pairwise genetic measure following Rousset’s genetic distance [*F*_*ST*_ (1 –*F*_*ST*_)^–1^] for a linear relationship using IBDws 3.15 [56]. Significance was assessed using the Mantel test with 10,000 permutations, and each axis was log-transformed before analysis.

## Results

### Phylogeographic analysis of mtDNA and microsatellites

**Fig 2:**
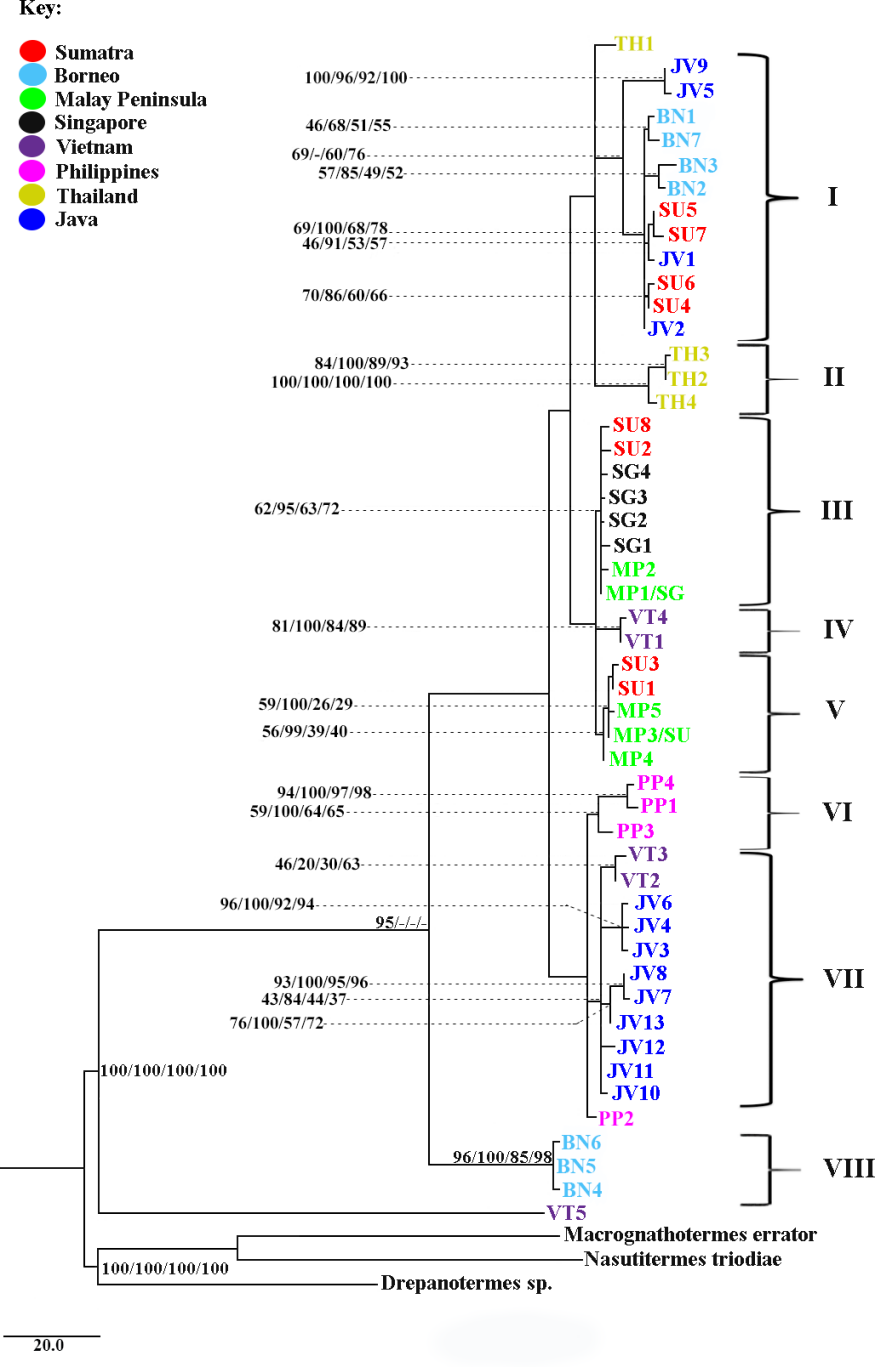
Maximum parsimony phylogram inferred from 49 concatenated 1,116 bp mtDNA haplotypes (see S1 Table for haplotype details on tip labels). The bootstrap values and posterior probabilities of the nodes were stated in the order of maximum parsimony, Bayesian, garli [31], and raxml [33]. Only nodes with values > 50% are shown. Roman numerals represent geographical clades inferred based on significant nodal support [58–59].

The concatenated sequence data for the mtDNA genes (1,116 bp) revealed 50 haplotypes with 98 variable sites from 141 individuals (out of 160) that produced amplifiable gene fragments for both the COII and 16S rRNA genes (S1 Table). Seventy-five of the variables sites were observed in more than one individual and therefore were phylogenetically informative. Transition to transversion bias was observed at a ratio of 5.027, which could be indicative of sequence saturation or recent divergence, among other possibilities [57]. Phylogenetic analyses of the mtDNA haplotypes using MP, two alternative ML estimates, and BI approaches produced congruent tree topologies. Eight major phylogenetic clusters were observed (clades I–VIII) (Fig 2). The Bornean termites (BN) were partitioned geographically into two clades: clade I involving termites from Sematan to Sibu and East Kalimantan located in the lower parts of Borneo and clade VIII involving termites from Miri, Bintulu and Sabah located in the upper parts of Borneo. Sumatran termites (SU) were also segregated into two distinct geographical clusters: termites from North Sumatra that forms an association with the Malay Peninsula (MP) populations in clades III and IV and a Riau-West Sumatra complex that forms an association with the West Java and Borneo populations in clade I. Javanese (JV) termites were clustered in clades I and VII, with no obvious pattern of haplotype segregation among localities within Java. Haplotypes from Thailand (TH) and the Philippines (PP) formed monophyletic groups in clades II and VI, respectively, although haplotypes TH1 and PP2 were unresolved. Positions of the Vietnamese haplotypes (VT) were poorly resolved and formed a paraphyletic assemblage on the phylogenetic tree.

Subsequently, we performed alternative phylogenetic hypothesis testing to validate the deep geographical separation observed among the Bornean and Sumatran termites, respectively. The statistical parameters AU, KH, SH, WKH, and WSH significantly rejected the monophyly of Sumatra and Borneo (i.e., alternative scenario, S6 Table) and thus showed support for the inferred phylogenetic split shown in Fig 2. A similar approach was used to test on whether the Vietnamese haplotypes could alternatively form a monophyletic group. All tests parameters failed to reject the hypothesis of Vietnam being monophyletic (S6 Table). This result suggests that an evolutionary relationship between haplotypes from Vietnam and Java, as observed in clade VII, may be present, but the relationship is poorly resolved. The discrepancy for the monophyly of Vietnam may also arise due to small sample size and the ambiguous position of haplotype VT5, which forms a sister group to all other *M*. *gilvus* (we noticed a similar condition in the divergence dating analysis; see S1 Fig). To avoid any spurious results due to the ambiguous status of VT5, we excluded this sample from subsequent analyses.

**Fig 3:**
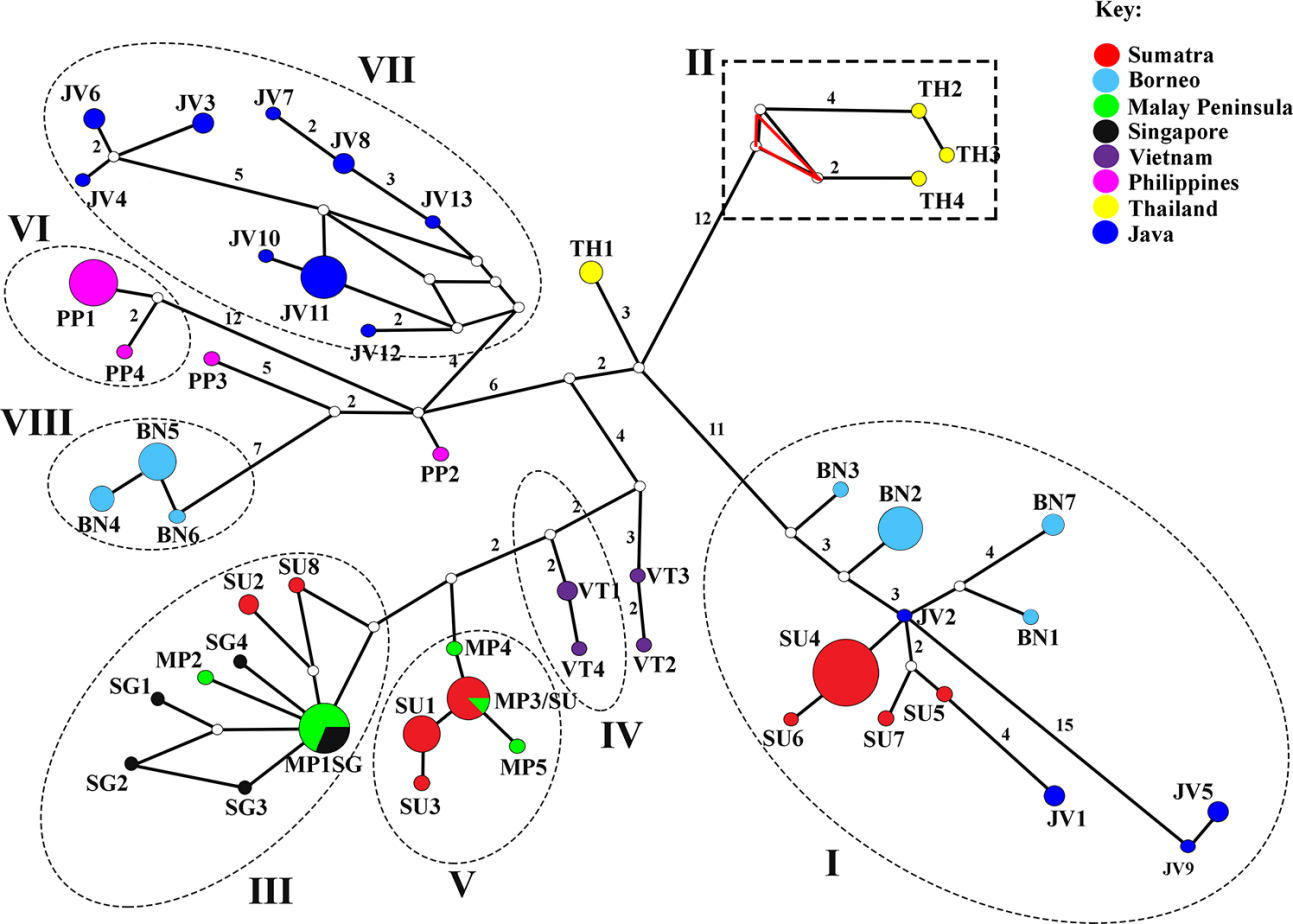
Minimum spanning network showing relationships among 49 mtDNA haplotypes based on 1,107 bp concatenated mtDNA sequences. Each circle denotes inferred haplotypes drawn to proportionate number of individuals. Haplotype codes and the number of individuals for each haplotypes are given in S1 Table. Numbers above branches indicate mutation steps between haplotypes. Open circles on the branches represent median vectors. Branches without numbers indicate a single mutation step. Roman numerals represent clades inferred by phylogenetic analysis. Note the deep divergence within the Borneo and Sumatra haplotypes.

The median-joining minimum spanning network of the termite mtDNA sequences displayed a distinct phylogeographic structure, with most haplotypes being regionally specific (Fig 3). Haplotype sharing was rare and restricted to geographically proximate localities, as demonstrated by haplotypes MP1/SG and MP3/SU that displayed close affinity between the Malay Peninsula with Singapore and Sumatra, respectively (see Fig 3). All Singapore haplotypes were derived from the Malay Peninsula lineage, as illustrated by the starburst pattern of haplotype MP1/SG. The absence of haplotypes with broad geographical distribution reflects a restricted gene flow across the geographical regions. A distinct population subdivision was observed within Borneo and Sumatra, which corroborated findings from the phylogenetic analysis. Haplotypes BN1-BN3 from Southwest Sarawak and haplotype BN7 from East Kalimantan shared a similar evolutionary pathway, with West Java’s JV2 being the centrum connecting these haplotypes. On the other hand, haplotypes BN4 and BN5 from Northwest Sarawak and BN6 from Sabah formed another distinct evolutionary group 29 mutation steps away from the former group. To ease interpretation in all subsequent analyses, we designated Southwest Borneo as B1 for the former group and Northwest Borneo as B2 (see Fig 1). In Sumatra, distinct population subdivision was observed between haplotypes from North Sumatra derived from the Malay Peninsula and haplotypes of the Riau-West Sumatra complex originating from West Java (Fig 3). An alternative statistical parsimony network generated using TCS 1.21 [36] resulted in a similar topology, albeit with minor differences. The exclusion of haplotypes TH3, TH4, and TH5 from northern Thailand because the 95% confidence level exceeded the parsimony connection limit of 14 as determined by the TCS program [36] (dashed box, Fig 3) and the presence of homoplasmy (red triangle, Fig 3) suggest that these haplotypes could be old or ancestral and had been long separated from the rest of the region.

**Fig 4:**
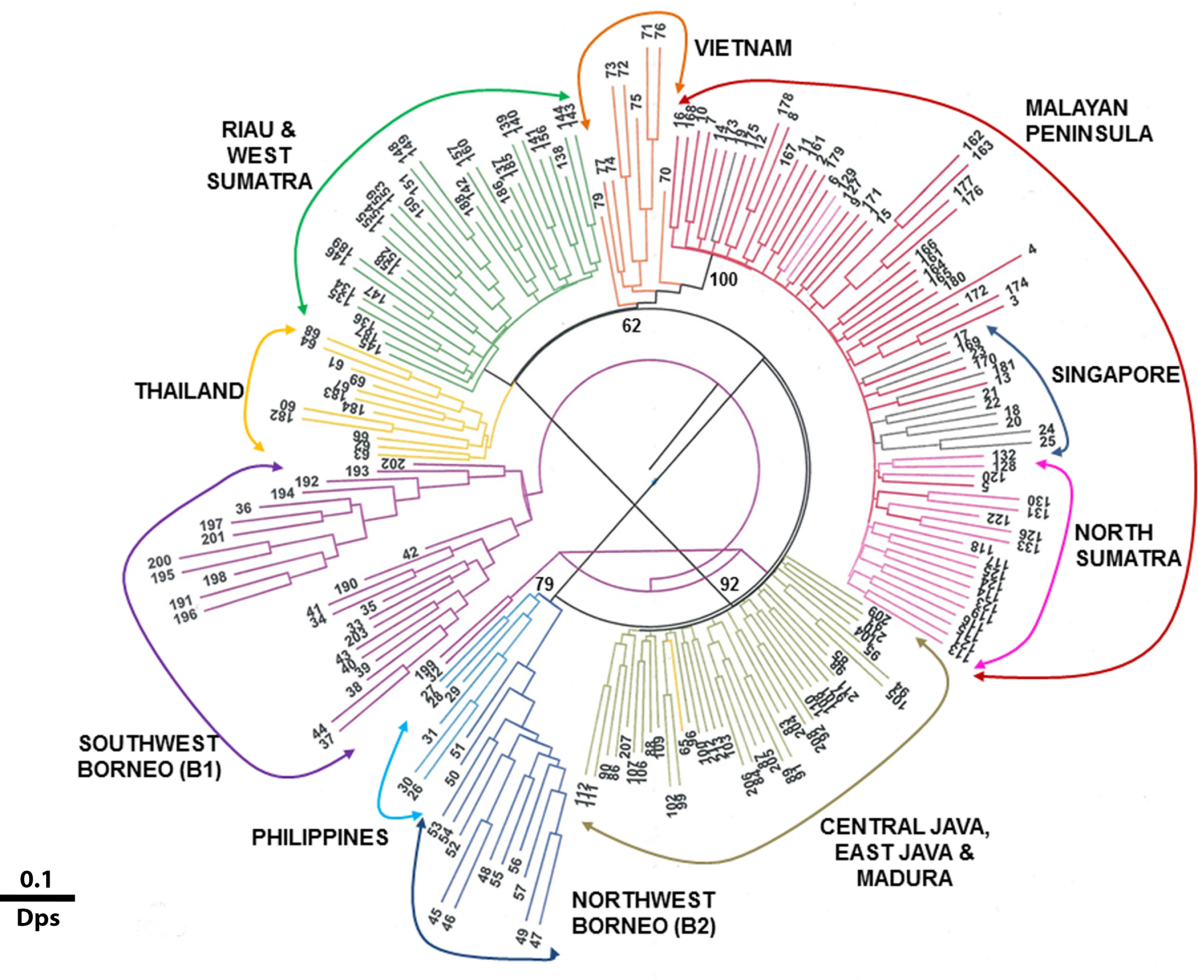
Phylogeographic relationship among 207 *M*. *gilvus* individuals based on composite microsatellite genotypes of 15 loci. Branches of the same colour represent termite individuals from the same geographical area. The NJ tree displayed here is based on Dps’ with the (1-ps) option in microsat [48]. The NJ tree based on the Dkf’ distance produced concordant topologies. Branch labels are individual sampling numbers (see S1 and S2 Tables). The bootstrap value for nodal support is indicated at the branches (only > 50% is shown) as inferred based on the NJ tree reconstruction in poptree2 [50].

A composite genotype from 15 *M*. *gilvus* specific microsatellite loci was obtained from 207 termite individuals. East Kalimantan (*n* = 2), West Java (*n* = 3), and one sample (V7) from Vietnam were excluded due to small sample size or poor amplification quality. A NJ analysis of individual termite genotypes based on the proportion of shared allele (Dps’) and kinship coefficient (Dkf’) genetic distances produced concordant topologies that support the population geographic subdivision observed in the mtDNA analysis (Fig 4). Termites from the Malay Peninsula were clustered together with samples from Singapore and North Sumatra (100% bootstrap support). The remaining *M*. *gilvus* genotypes were partitioned into six clearly differentiated clusters (Fig 4). Borneo samples were split into B1 (Southwest Borneo) and B2 (Northwest Borneo) clusters that were identical to those of the mtDNA analysis, except for the exclusion of the East Kalimantan sample from B1 and with the Philippines being a sister group to B2 (78% bootstrap support). The Indochinese subcontinent was separated into a single group of termites from Thailand (except sample no. 16) and an independent assemblage of the Vietnam samples. The remaining samples from Riau and West Sumatra collectively formed a single cluster, and all of the Javanese samples were clustered into another group (78% bootstrap support).

### Genetic variation and molecular diversity

Quantitative estimates of mtDNA diversity recorded a wide range of genetic variation within each termite population, with an overall haplotype diversity (*H*_*d*_) of 0.31 (Table 1). Several regions showed extremely low or high genetic diversity. The Indochinese populations (Thailand and Vietnam) had the highest *H*_*d*_ index and thus were most likely ancestral, whereas the Sumatran termites were the least genetically diverse and therefore may have been recently introduced or experienced ancestral genetic bottleneck. Similarly, the Bornean termites displayed an unexpectedly low *H*_*d*_ of 0.27 given the extensive sampling and effective sample size (Fig 1). Overall, genetic diversity assessment consistently showed that Thailand and Central Java harbored the highest genetic variation across various diversity measures (see Table 1). Additionally, a marked increase in the sequence divergence among individuals within Sumatra and Borneo (π > 1.0%) followed by a substantial gain in θ_S_ values (bold faced, Table 1) validates the observed population subdivision within these regions. The neutrality test displayed no significant departures from expectations in any population at α = 0.01 (Table 1).

**Table 1:** Estimates of molecular genetic variation from combined mtDNA sequences (1,107 bp).

Populations	*n*	No. of haplotypes	No. of substitution sites	No. private substitution sites	Haplotype diversity (*Hd*)	Nucleotide diversity (π) (averaged over loci)	Theta (θs)	Tajima's D	Fu's Fs	Nucleotide diversity (π)
Malay Peninsula	14	5	6	1	0.36	0.00149	1.89	–0.47	–0.42	1.64
										
Singapore	8	5	4	3	0.63	0.00123	1.54	–0.52	–2.04	1.36
										
The Philippines	6	4	15	10	0.67	0.00610	6.57	0.09	2.39	9.13
										
Java	28	13	44	16	0.46	0.01094	11.31	0.19	1.97	13.11
West Java	3	2	3	0	0.67	0.00182	2.00	0.00	2.36	3.33
Central Java	10	7	34	17	0.70	0.01420	12.02	1.35	2.09	16.51
East Java	8	3	6	0	0.38	0.00186	2.31	–0.56	1.61	2.04
Madura	7	3	4	2	0.43	0.00104	1.63	–1.43	0.26	1.14
										
Sumatra	48	9	31	6	0.17	**0.01059**	**6.99**	2.18	10.79	13.03
North Sumatra	21	5	8	2	0.24	0.00182	2.22	–0.33	0.77	2.00
Riau	14	3	5	1	0.21	0.00065	1.57	–1.89	0.18	0.71
West Sumatra	13	2	4	1	0.15	0.00056	1.29	–1.77	1.47	0.62
										
Indochina	11	9	33	17	0.82	0.01211	11.67	0.63	0.52	14.69
Thailand	5	4	21	16	0.80	0.01124	10.08	1.56	2.04	13.00
Vietnam	6	5	10	3	0.83	0.00493	4.80	0.89	0.61	5.40
										
Borneo	26	7	31	8	0.27	**0.01205**	**8.12**	2.27	10.17	15.42
B1	13	3	10	2	0.23	0.00152	3.22	–1.93	1.96	1.67
B2	11	3	2	1	0.27	0.00056	0.68	–0.29	–0.31	0.62
East Kalimantan	2	1	23	2	0.50	–	–	–	–	–

Similar to the mtDNA results, overall microsatellite diversity was highest in Thailand with an average *H*_E_ of 0.726 and allelic richness of 4.88 followed by populations from Java and Vietnam (Table 2). Eleven of the 13 geographical groups showed population-specific alleles that tended to represent the extreme sizes of allele distributions (S8 Table). Of the 42 private alleles, 17 were either the smallest or the largest size class among all groups, and 24 were either the smallest or largest for a specific population, suggesting a recent derivation. The number of alleles per locus across all populations ranged from 8 to 21 alleles, indicating a modest level of polymorphism (S8 Table). Only 5 of the 13 populations displayed significant departures from HWE at several microsatellite loci (Table 2). This deviation was most substantial in the Malay Peninsula and B1 populations. This was associated with a significant heterozygosity deficit, which could be a result of considerable inbreeding within these populations (*F*_*IS*_ = 0.318 in B1; *F*_*IS*_ = 0.199 in the Malay Peninsula). Furthermore, assessment of invariant loci (monomorphic loci) revealed that the Malay Peninsula, Singapore, and North Sumatra populations shared a monomorphic locus MG3, whereas Riau, West Sumatra, Central Java, and East Java were monomorphic at locus MG2 (Table 2). These results indicate a close genetic association among these regions, which supports the results of the mtDNA analyses.

**Table 2:** Genetic variation across 15 microsatellite loci in *M*. *gilvus* populations.

Pop	*n*	Average observed heterozygosity (H_O_)	Average expected heterozygosity (H_E_)	Locus with significant departure from HWE^a^	Monomorphic locus	Average FIS value per locus	Average Allele Size range (repeats per locus)/ Average allelic richness (A_R_)	G-W Index
PM	36	0.441	0.549	MG5,MG11,MG9,MG36	MG3	0.199	5.29/2.88	0.81
SG	10	0.554	0.486	-	MG3,MG7	-0.149	2.92/2.31	0.81
TH	13	0.643	0.726	MG36	-	0.120	6.07/4.55	0.88
VT	9	0.507	0.686	MG7,MG5	MG36	0.273	5.07/3.89	0.89
NS	21	0.376	0.405	-	MG3	0.075	2.64/2.17	0.82
RI	19	0.593	0.588	-	MG2	-0.008	4.21/3.07	0.87
WS	13	0.495	0.556	-	MG2	0.114	3.50/2.81	0.82
CJ	14	0.448	0.590	-	MG2	0.248	5.21/3.30	0.83
MD	16	0.618	0.641	MG34	-	0.037	6.27/3.94	0.83
EJ	10	0.592	0.695	-	MG2,MG9	0.157	5.62/3.77	0.84
B1	27	0.282	0.412	MG2,MG37,MG5,MG11,MG36	MG1,MG6,MG9	0.318	4.00/2.16	0.80
B2	13	0.522	0.502	-	MG1	-0.039	2.93/2.39	0.83
PP	6	0.488	0.658	-	MG33	0.278	4.07/3.57	0.81

Key: PM- Malay Peninsula, SG-Singapore, TH-Thailand, VT-Vietnam, NS-North Sumatra, WS-West Sumatra, RI-Riau, CJ-Central Java, EJ-East Java, MD-Madura, PP-the Philippines, B1-southwest Borneo, B2-northwest Borneo

^a^ after Bonferroni adjustment at significance level = 0.05

Tests of null alleles and linkage disequilibrium (LD) displayed an inconsistent pattern and often associated with significant departures from HWE within the respective population. This suggests that either the presence of null alleles or a significant LD could be the result of local sampling effect in the respective populations rather than non-random association of alleles between the loci. There were no scoring errors due to stutters or large allelic drop out detected from this analysis. All populations in this study displayed a relatively high Garza-Williamson index (*P* > 0.80), indicating that the populations were not experiencing recent bottleneck effect (Table 2).

### Genetic differentiation

Pairwise genetic differentiation between population pairs broadly supported the observed phylogeographic structure of *M*. *gilvus* across SEA. The global *Φ*_*ST*_ index (weighted average of population specific *Φ*_*ST*_ values) was remarkably high at 0.831 (*P* < 0.0001), indicating profound genetic differentiation among sites. Unexpectedly, geographically distant population pairs such as Thailand and the Philippines as well as Vietnam and Java displayed close genetic association with no significant genetic differentiation (*Φ*_*ST*_ bold faced, S7 Table). In contrast, the geographically close B1 and B2 populations of Borneo displayed significant genetic differentiation (*Φ*_*ST*_ = 0.950; *P* < 0.0001) despite the absence of any obvious geophysical barriers separating them. Similarly, North Sumatran termites were genetically isolated from the Riau and West Sumatran termites, with a high *Φ*_*ST*_ > 0.930; *P* < 0.0001. These conditions further suggest that the genetic association between sites was not restricted to contemporary processes but rather was influenced by past complex historical processes that took place in SEA.

For microsatellites, all pairwise *F*_*ST*_ comparisons also revealed significant genetic differentiation except for three geographically proximate pairs: West Sumatra-Riau, East Java-Central Java, and East Java-Madura (S9 Table). Genetic differentiation was most substantial between population pairs from Borneo and Sumatra suggesting restricted contemporary gene flow between these regions. In fact, the Bornean termites were markedly differentiated from the rest of the mainland SEA populations (*R*_*ST*_ > 0.60 among all pairs). Similar to the mtDNA findings, unexpectedly high pairwise *F*_*ST*_/*R*_*ST*_ estimates were found among population subdivisions within Borneo and Sumatra (S9 Table). The Mantel test revealed a significant isolation-by-distance pattern between geographic distance and genetic distance for both mtDNA (*r* = 0.371, *P* < 0.05) and microsatellite analysis (*r* = 0.473, *P* < 0.001).

### Genetic structure

**Fig 5:**
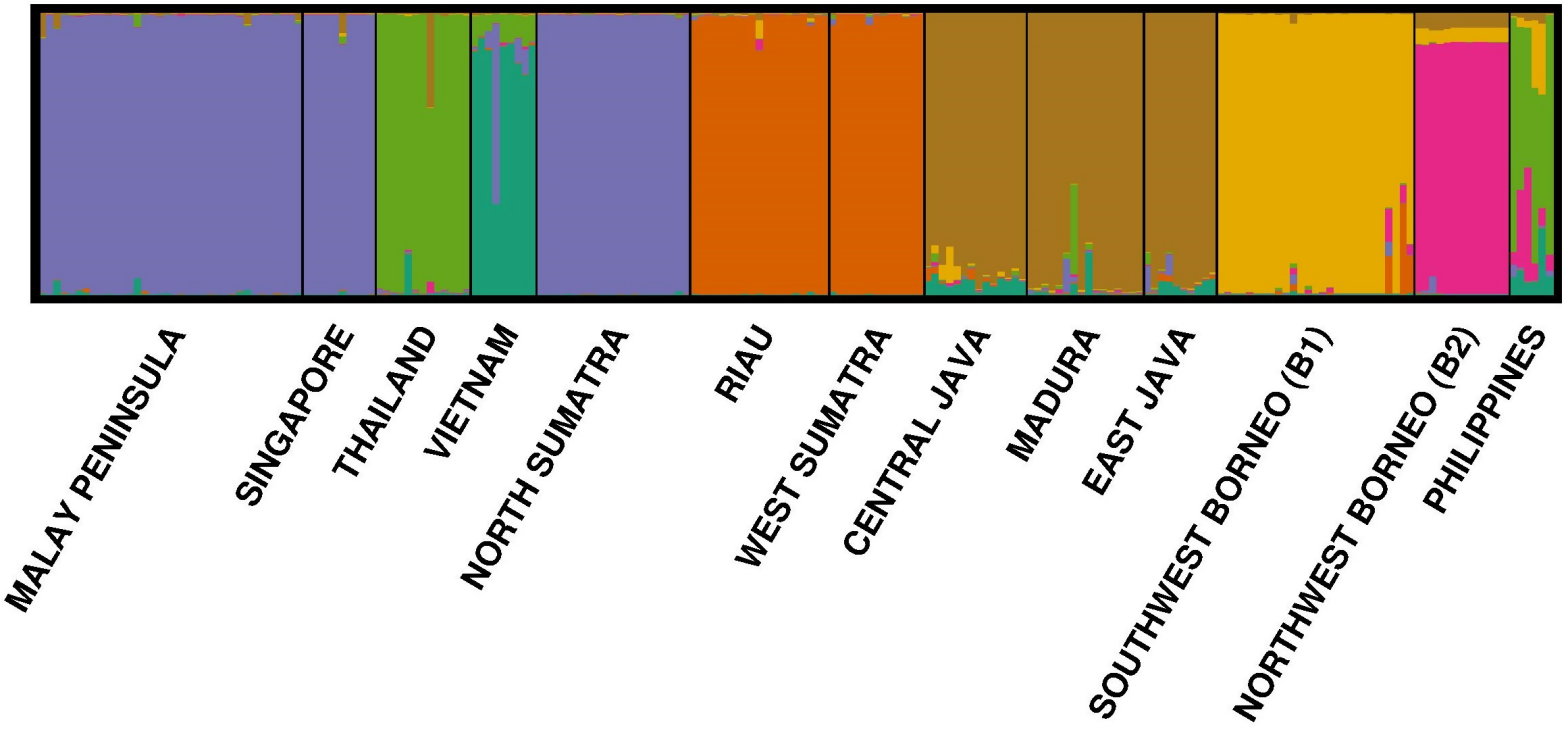
Membership coefficient bar plot displaying population clusters (*k* = 7) inferred from structure [51] analysis. Vertical line denotes probability of individual assignment to the respective cluster. Each bar denotes each individual genotyped from each population. Cluster memberships are represented by colour as visualised from the program distruct [53].

An assignment test based on the Bayesian clustering of multilocus microsatellite genotypic data as implemented in the program structure [51] supported the partitioning of the termite individuals into seven distinct clusters (ΔK = 7, Fig 5) as observed from the NJ tree with one exception. In this analysis, the termites from the Philippines were clustered with the Thailand group instead of forming an association with B2. To validate the clustering accuracy, the genotypic data from each termite were assigned back into the respective clusters as determined by structure [51]. We found that 99.6% of the total individuals were correctly assigned to the respective clusters with MIGPRIOR option set at 0.01. Only one individual sample from Vietnam (colony V6) showed significant probability of being a migrant (*P* < 0.001) that may have had recent ancestry in the Thailand-Philippines cluster; the probabilities were 0.005 and 0.769 that the individual came directly from this group or had a single ‘parent’ from this population, respectively.

### Spatiotemporal dynamics of *M*. *gilvus* dispersal in SEA

**Fig 6:**
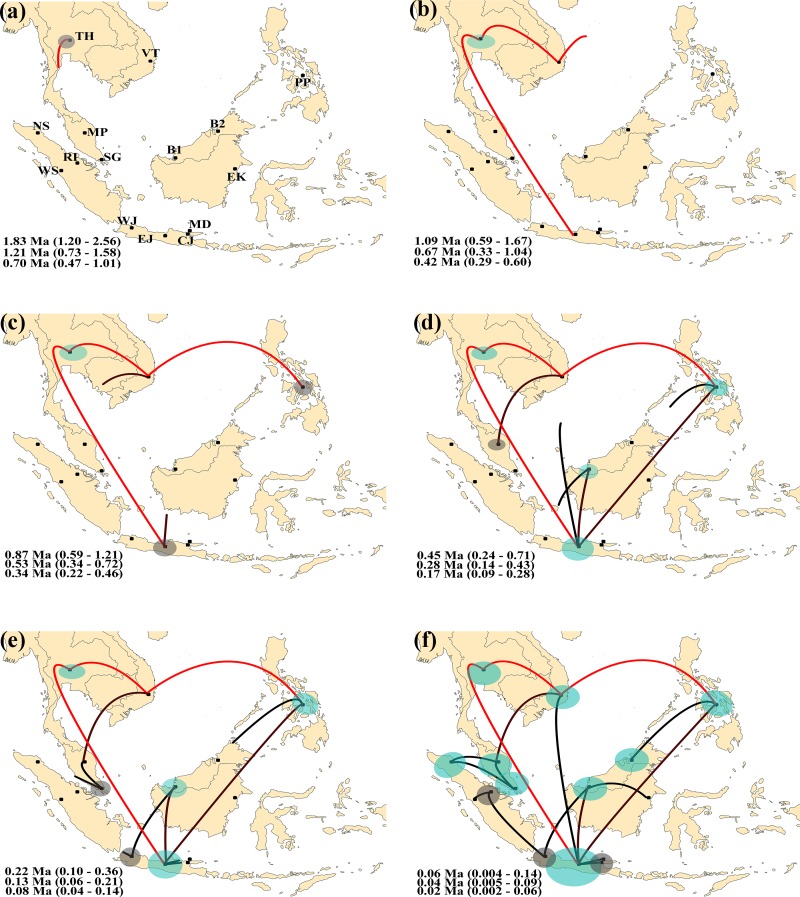
Ancestral reconstruction of *M*. *gilvus* dispersal over the last two million years across Southeast Asia inferred from beast [39]. The different panels represent temporal projections of the reconstructed migration events that have occurred up to present day in chronological order (a-f). Link heights indicate relative durations of the branches upon which the inferred migration occurred, and the red–black colour gradient illustrates relative age of transition (older–recent). Circles represent ancestral population size, and the deep–faint blue colour gradient represents age (older–recent). Age estimates of the migration events for each projection are given in the order of mtDNA age calibrations from top to bottom: 0.9% Myr^–1^, 1.5% Myr^–1^, and 2.28% Myr^–1^. To comply with PLoS ONE submission requirements for figures under the CC BY license, a replacement figure has been provided here. This figure is similar but not identical to the original image, and is therefore for illustrative purposes only. The original image will be provided upon request from the authors. Key: MP-Malay Peninsula, SG-Singapore, TH-Thailand, VT-Vietnam, NS-North Sumatra, WS-West Sumatra, RI-Riau, CJ-Central Java, EJ-East Java, WJ-West Java, MD-Madura, PP-the Philippines, B1-Southwest Borneo, B2-Northwest Borneo, EK-East Kalimantan.

Ancestral reconstruction through discrete phylogeographic analysis in beast [39] revealed a pattern of historical migration of *M*. *gilvus* among study sites across SEA. Thailand was supported as the root location for all lineages across SEA with the highest root probability at 95% Highest Posterior Density (HPD) at 0.2076, and the root height of the current dataset estimated at 1.83 Ma (95% HPD: 1.20–2.56 Ma), 1.21 Ma (1.58–0.73 Ma), and 0.70 Ma (0.47–1.01 Ma) based on the 0.9% Myr^–1^, 1.5% Myr^–1^, and 2.28% Myr^–1^ of mtDNA substitution rates, respectively. In the absence of independent criteria to select between the different time estimates, the age estimate using the 1.5% Myr^–1^ divergence rate was selected as a rough median to illustrate the time dynamics of *M*. *gilvus* divergence, but the estimates obtained with the other calibration ages are also shown (Fig 6).

Under this scenario, the first migration event involved a bidirectional movement of termites eastwards to Vietnam and southwards to central Java, where their respective ancestral populations were founded around 0.67 Ma (0.33–1.04 Ma) (Fig 6B). Eastward migration extended further until it reached the modern Philippines around 0.53 Ma (0.34–0.72 Ma) (Fig 6C). A single colonization event from Vietnam into Malaysia took place around 0.31 Ma (0.16–0.49 Ma) and was followed closely by a migration event from central Java into B1 in Borneo at 0.28 Ma (0.14–0.43 Ma) (Fig 6D). A West Java lineage originated from B1 around 0.14 Ma (0.05–0.23 Ma) (Fig 6E), and subsequently the B2 lineage was established independently much later around 0.06 Ma (0.01–0.12 Ma) from the Philippines. The North Sumatran termites derived from the Malay Peninsula lineage following two migration events between 0.04 Ma (0.005–0.09 Ma) and 0.02 Ma (0.0004–0.05 Ma) (Fig 6F). Concurrently, another independent lineage of Sumatran termites originating from West Java emerged at Riau and subsequently in the adjacent West Sumatra about 0.05 Ma (0.007–-0.11 Ma) (Fig 6F). The termites from central Java eventually spread out to establish the East Java and Madura lineages around 0.09 Ma (0.02–0.16 Ma). East Kalimantan termites emerged last in the analysis and originated from the B1 lineage.

Fossil calibration dated the origin of the fungus-growing termites and the divergence of the genus *Macrotermes* at 21.5 Ma (11.6–38.2 Ma) and 14.4 Ma (7.4–26.0 Ma), respectively. These results are consistent with the previous estimates given by Nobre et al [46] of 31.0 Ma (16.7–48.8 Ma) and 16.1 Ma (9.0–25.0 Ma). The *Macrotermes gilvus* clade was estimated to have diverged at 2.67 Ma (1.2–5.32 Ma) (S1 Fig) from *M*. *annandalei* and *M*. *chaiglomi* of Asia. The branching pattern within the *M*. *gilvus* clade (S1 Fig) is generally congruent with the tree topology observed in Fig 2 and the fossil calibration method supported the time dynamics from the discrete phylogeographic analysis, as the divergence of *M*. *gilvus* in SEA took place within the Pleistocene epoch (~2.00–0.01 Ma).

## Discussion

The patterns of spatial structure resolved from mtDNA and microsatellite data were mostly consistent in displaying a highly structured *M*. *gilvus* populations in SEA. Eight phylogeographic groups were identified in this study based on phylogenetic relationships, genetic structure, and molecular diversity indices at both markers: Thailand, Vietnam, the Malay Peninsula/Singapore/North Sumatra, Riau/West Sumatra, Java, Southwest Borneo (B1), Northwest Borneo (B2), and the Philippines. The global *F*_*ST*_/*R*_*ST*_ value at 0.34/0.64 was highly significant (*P* < 0.0001), signaling a substantive genetic differentiation among the populations as a whole, and most of the pairwise comparisons displayed a pronounced level of genetic differences (> 0.25). Recent gene flow and haplotype sharing between the phylogeographic groups were rare, which corroborates this finding. Nevertheless, the genetic similarity and haplotype sharing between North Sumatra and the Malay Peninsula (MP3/SU) suggests possibilities for retention of ancestral traits and/or recent gene sharing across these two populations.

Regression of genetic distance against geographical distance revealed a significant positive isolation-by-distance pattern for both mtDNA and microsatellite data. This indicates that the present day South China Sea may in fact act as an effective barrier to dispersal among the insular termites (i.e., Borneo, Sumatra, Java, and the Philippines) and the termites from mainland SEA. However, the correlation coefficient values from both molecular markers were weak (*r*^*2*^ < 0.22), suggesting that the genetic differentiation between populations was not a simple function of genetic isolation-by-distance but rather a complex interaction of other historical processes followed by an isolation mechanism. This is plausible given the complexity of geological perturbations taking place surrounding SEA in the past, including repeated sea level fluctuations [4], considerable tectonic activity [60], and associated climatic and vegetation changes [11, 20]. The absence of a recent bottleneck event and an overall high average *H*_E_ from microsatellite data along with neutral expectation following mtDNA’s Tajima’s D and Fu’s *Fs* tests indicate that each studied population is demographically stable, mixing randomly, and potentially in mutation-drift equilibrium. The average low inbreeding coefficient in all populations corroborates the occurrence of random mating and potentially reflects a substantial effective population size.

Dating phylogenies remains an uncertain process, as estimated divergence times vary with DNA regions, calibration procedures, and fossil calibration points. Nevertheless, the credibility intervals of the different time estimates used in the present study often overlapped, and the dispersal events were consistently placed within the Pleistocene time frame. This result is in line with the belief that climatic fluctuations of the Pleistocene were a vital driving force for genetic divergence and speciation [61]. Our finding based on the discrete phylogeographic analysis has provided novel insight on the dispersal framework of early faunal migration associated with open vegetation in Pleistocene SEA. The dispersal pattern was multidirectional, and Thailand was predicted as the ancestral root of *M*. *gilvus* radiation in SEA. This finding is congruent with previous studies on a northern Southeast Asian origin of tephritid fruit fly in this region [41, 62, 63].

We identified at least three major range expansions of *M*. *gilvus* from this study: 1) 1.09–0.42 Ma (early Pleistocene)–migration of termites due east and south from Indochina to establish the Java and the Philippines lineages, respectively, 2) 0.45–0.17 Ma (mid Pleistocene)–establishment of the Malay Peninsula and Southwest Borneo lineages from Vietnam and Central Java, respectively, and 3) 0.06–0.02 Ma (late Pleistocene)–independent colonization of Sumatra from the Malay Peninsula and West Java, respectively. The establishment of the Javanese lineage from Thailand (Fig 6B) provides important evidence for the presence of an open corridor connecting Indochina to Java during the Pleistocene. This finding agrees in part with the presence of the north-south savanna corridor proposed by Heaney [9] (see Fig 1). Following Heaney’s proposal, the migration of savanna-dependent species would have commenced in Indochina and progressed through the Malay Peninsula before reaching insular SEA. Spatiotemporal patterns from our study, however, showed that an inland dispersal route developed first, long before it expanded into the eastern margin of the Malay Peninsula. The invasion of *M*. *gilvus* from Vietnam into the Malay Peninsula approximately 0.31 Ma (95% HPD: 0.16–0.49), after its dispersal to Java 0.67 Ma (95% HPD: 0.33–1.04) (Fig 6D), illustrates this scenario. This mode of dispersal corresponds to the presence of the oldest hominin fossil in the central Java region at ca. 1.6–1.02 Ma [19] before other insular habitats of SEA, which supports the existence of a dispersal corridor that allowed the migration of terrestrial fauna (including *Homo erectus*) from Asia through Sundaland into Java well into the Pleistocene [9, 11].

For the first time, we hypothesize the presence of a west-east dispersal corridor stretching from mainland SEA across Sundaland towards the Philippines during the Pleistocene. Today, the northern boundary of Sundaland is hard to delineate because the region is now mostly submerged [11], which makes it difficult to determine the type and extent of vegetation (if any) necessary for the west-east migration of terrestrial species from mainland SEA. However, discrete phylogeographic analysis provided evidence for an effective termite migration event taking place from Indochina to establish new colonies in the east (Fig 6C). In addition, the microsatellite genotypic data from structure [51] analysis revealed a significant close association between the Philippines and Thailand despite physical isolation by the present-day South China Sea (Fig 5). The *M*. *gilvus* population from the Philippines is genetically similar to the Thailand and Vietnam populations as compared to other regions (even Borneo, which is geographically closer to the Philippines; *F*-statistics, S7 Table). In fact, the three populations displayed no significant genetic differentiation from one another.

Schutze et al [41] reported that a similar eastward movement of tephritid fruit flies from Thailand to the modern Philippines took place approximately 360 ka, which further supports the existence of an effective dispersal across the South China Sea. Although natural dispersal mode of *M*. *gilvus* does not favour transoceanic dispersal, long-distance dispersal of terrestrial organisms via rafting on vegetation mats is a possibility [64]. Besides *M*. *gilvus*, there are many other soil-dependent termite taxa in Sundaland including the fungus feeding genus *Odontotermes* and soil feeders like *Pericapritermes*, *Discupiditermes*, *Coxocapritermes* as well as many nasute genera [65, 10]. These rainforest taxa probably had even more restrictive over-water dispersal requirements than *M*. *gilvus*, however, various species from the genera *Odontotermes*, *Pericapritermes*, *Bulbitermes*, *Grallotermes*, *Hospitalitermes*, and *Lacessitermes* are common to the Philippines. Their presence necessitates over water rafting over very narrow Pleistocene seas or possibly larger gaps during interglacial high sea stands. Moreover, Nobre et al. [66] show that the *Macrotermitinae* (fungus-growing termite family) overcame a wide 300+ km Miocene oceanic gap between Africa and Madagascar. Since evidence for a direct land connection between Indochina and the Philippines is still lacking, under this scenario, over water dispersal mechanism would be most likely explanation for *M*. *gilvus* to have dispersed across these two regions.

We found no evidence of effective migration events between Sumatra and Borneo. The high genetic divergence between the two regions is congruent with results of other studies on speciation in neotenic net-winged beetles [67] population structure of the striped snakehead fish [68], and also as displayed by the dispersal pattern of the tephritid fruit fly [41], suggesting that the two regions have restricted dispersal event for selected taxa despite both landmasses were joined numerous times during the Pleistocene due to shallow waters (46m) of the Java sea. Slik et al. [16] show that there was no evidence for a savanna corridor between Borneo and Sumatra, but show evidence for coarse soil barrier of exposed sea floor. The soil condition might be deemed unsuitable for *M*. *gilvus* to disperse across these two regions during the Pleistocene. Instead, central Java displayed remarkable habitat stability (i.e., pronounced genetic diversity), serving as a central link to the south of the Equator and radiating out to establish termite lineages in Southwest Borneo, West Sumatra, and East Java. Our findings support the hypothesis of a minimal savanna corridor [11] that would allow dispersal of open vegetation adapted species between Java and Borneo. The data provide no support for the presence of a broader maximal savanna corridor [11] that would allow mixing of populations between Sumatra, Java, and Borneo. A land bridge may have connected the insular environments during the LGP, but it did not persist for long enough and/or the vegetation type did not allow effective migration to take place between Sumatra and Borneo.

Based on the termite dispersal events, we suggest two alternative routes of entry by which the termite could have colonized insular Sumatra as inferred from the discrete phylogeographic analysis. The first route involves a northern incursion from the southwest region of the Malay Peninsula and Singapore across the Malacca Straits anytime between 60 and 20 ka. The timing of the dispersal events suggests that colonization of *M*. *gilvus* occurred soon after the catastrophic super-eruption of Toba approximately 73.5 ka in northern Sumatra [69]. Such a post Toba-eruption re-colonization has been previously suggested for several mammals species in Sumatra [70–73]. The aftermath may have provided a suitable habitable environment to develop in the lowland areas surrounding the caldera. Geological records associated with ash layers attributed to the Toba eruption [74–75] suggest the possibility of an open vegetation phase of landform development related to the LGP in the southwest Malay Peninsula. Similarly, pollen records support the premise that the vegetation during the penultimate glacial maximum (~195–128 ka) near Kuala Lumpur was an open savanna dominated by pines and grasses [76]. The timing and the location of this open vegetation phase fits the inferred dispersal route presented in this study, suggesting that a stretch of savanna corridor across the Malacca Straits would have allowed effective migrations to take place. Our study suggests that the dispersal event existed after the Toba explosion, but it is possible that pre-existing termite populations in North Sumatra resulting from an earlier migration event could have been erased by the Toba event.

The second route involves the incursion of termites in the south originating from Western Java, spreading to Riau Province, and subsequently residing in West Sumatra. Despite an ongoing debate about the timing of the emergence of the modern Sunda Straits (see Fig 1) that separate Java and Sumatra (5^th^– 6^th^ century AD ([77–78] or 56–74 ka ([11, 79]), our results show that termite migration took place between Java and Sumatra sometime between 60 and 20 ka (based on all time estimates; Fig 6F). This suggests that a land bridge connecting these areas was present at that time. Alternatively, if the Sunda Straits did exist by this time, transoceanic dispersal would have been required to traverse the two islands. To our knowledge, this is the first time the occurrence of such a migratory route has been proposed based on phylogeographic evidence. On the other hand, the two disjunct *M*. *gilvus* populations in Sumatra could have directly resulted from the massive impact of super-eruption of Toba that could have splintered the populations sufficiently to enhance haplotype divergence as both populations share similar timing for colonization event to take place.

An unexpected genetic break was observed between B1 and B2 in Borneo despite the absence of contemporary geophysical barriers separating them. Genetic isolation-by-distance is possible, but we observed contrasting scenario based on the genetic affinity shared between the Malay Peninsula and North Sumatra termites, which are separated by a similar geographical distance as seen between B1 and B2 (~150 km). Alternatively, unfavorable soil condition between the two habitats may play a role as barriers to dispersal but this feature remains to be investigated. It is also important to note that B1 and B2 termites displayed different evolutionary origins, and the time dynamics of their colonization events were hundreds of thousands years apart. This finding suggests multiple points of entry for ancient terrestrial fauna to colonize Borneo at different time points during the Pleistocene glacial phase.

This study has provided insight about the biogeography of SEA and the relative impacts of palaeoenvironmental changes in shaping the distribution and dispersal of local taxa during the Pleistocene glacial periods. Different dispersal events and genetic isolation have shaped the present day biogeography of *M*. gilvus in this region. Our findings support several existing hypotheses of dispersal corridors in SEA and additionally suggest an alternative dispersal routes for terrestrial fauna to disperse across Sundaland during the Pleistocene. Despite several attempts to simulate the vegetation distribution at the last glacial maximum based on climatic/vegetation modelling (e.g., [80]), evidence of the extent to which a forest or a continuous/broken savanna corridor covered parts of the core of Sundaland remains sparse and conflicting [11]. In any case, our study suggests that effective dispersal events did exist on Sundaland at various points in time enabling dispersal of soil-dependent terrestrial fauna in Pleistocene SEA.

## Supporting information

S1 Table Sampling details of *M*. *gilvus* across SE Asia. Positive PCR amplifications are indicated with x symbol.
(DOCX)

S2 Table Additional *M*. *gilvus* samples used in microsatellite analysis. (DOCX)

S3 Table Best-fit nucleotide substitution model based on hierarchical likelihood ratio test (hLRT) and Akaike’s information criteria (AIC). (DOCX)

S4 Table Partitioning schemes for the concatenated dataset based on GARLI and MrBayes optimization process. Numbers in bracket represents 1^st^, 2^nd^ and 3^rd^ codon positions of COII gene respectively. Partitioning scheme with best AIC score is highlighted in bold.
(DOCX)

S5 Table Bayes factor analysis. Best partitioning scheme for Bayesian inference is shown in bold. Italicized values indicate number of parameters used to calculate the bayes factor.
(DOCX)

S6 Table Maximum likelihood values and statistics for the alternative phylogenetic hypotheses calculated in GARLI and CONSEL. (DOCX)

S7 Table Pairwise Φ_ST_ values (lower diagonal) and geographical Euclidean distance (shortest distance between pairs of populations, km) used in isolation-by-distance analysis (upper diagonal). (DOCX)

S8 Table Allelic richness (A_R_), number of alleles (N_A_), number of private alleles (in parenthesis) and its percentages (P_A_) for each locus and population. (DOCX)

S9 Table Pairwise R_ST_ (sum of squared sized differences–lower diagonal) and F_ST_ (number of different alleles–upper diagonal) generated from Arlequin 2.0. (DOCX)

S1 Fig Divergence dating of the fungus-growing termites based on COII gene. (DOCX)

S1 Appendix PCR conditions and primers used for the mtDNA sequencing. (DOCX)

S2 Appendix Permission to reprint copyrighted figure. (DOCX)
