# Supplementary material for: Phylogeography of the termite Macrotermes gilvus and insight into ancient dispersal corridors in Pleistocene Southeast Asia
Source: PLoS One. 2017 Nov 29;12(11):e0186690. doi: 10.1371/journal.pone.0186690 (PMC5706666; doi:10.1371/journal.pone.0186690)
Supplement: S1 Table — Positive PCR amplifications are indicated with x symbol. (DOCX) [file pone.0186690.s001.docx]

**S1 Table. Sampling details of *Macrotermes gilvus* across SE Asia.** Positive PCR amplifications are indicated with x symbol.

| **No** | **Code** | **Haplotypee** | **Date** | **Localities** | **Latitude** | **Longitude** | **COII** | **16S** | **Both** |
| --- | --- | --- | --- | --- | --- | --- | --- | --- | --- |
|  | **MALAYAN PENINSULA [*n* = 16, *Ne* = 14, H = 5, H_­­­­­d­_ = 0.36]** | | | | | | | | |
| 1 | GG1 | MP1/SG | Jul-10 | Gelugor, Penang | N 05° 22.007 | E 100° 18.632 | x | x | x |
| 2 | BL 1 | MP1/SG | Jul-10 | Bayan Lepas, Penang | N 05° 19.228 | E 100° 17.381 | x | x | x |
| 3 | TB6 | MP2 | Jul-10 | Teluk Bahang, Penang | N 05° 27.607 | E 100° 12.314 | x | x | x |
| 4 | 199_1 | MP1/SG | Jul-10 | USM, Penang | N 05° 21.741 | E 100° 18.390 | x | x | x |
| 5 | KULa | - | Jul-10 | Kulim, Kedah | N 05° 23.413 | E 100° 33.767 | x |  |  |
| 6 | KULc | - | Jul-10 | Kulim, Kedah | N 05° 23.457 | E 100° 33.735 |  | x |  |
| 7 | AS | MP1/SG | Jul-10 | Alor Setar, Kedah | N 06° 07.174 | E 100° 22.026 | x | x | x |
| 8 | SP a | MP1/SG | Jul-10 | Sungai Petani, Kedah (Taman Rekreasi) | N 05° 38.238 | E 100° 29.078 | x | x | x |
| 9 | Kel 2 | MP1/SG | Jul-10 | Tanah Merah, Kelantan | N 05° 48.458 | E 100° 09.065 | x | x | x |
| 10 | Kel 4 | MP1/SG | Jul-10 | Tanah Merah, Kelantan | N 05° 48.416 | E 100° 08.999 | x | x | x |
| 11 | 002 p | MP1/SG | Nov-10 | Lenggong,Perak | N 05° 07.626 | E 100° 59.522 | x | x | x |
| 12 | 004 p | MP1/SG | Nov-10 | Lenggong,Perak | N 05° 07.571 | E 100° 59.375 | x | x | x |
| 13 | SERD | MP3/SU | Nov-10 | Serdang, Selangor | N 02° 59.542 | E 101° 42.626 | x | x | x |
| 14 | TER | MP4 | Nov-10 | Kuala Berang,Terengganu | N 05° 04.098 | E 102° 59.918 | x | x | x |
| 15 | NS | MP1/SG | Dec-10 | Rembau, Negeri Sembilan | N 02° 35.141 | E 102° 06.004 | x | x | x |
| 16 | JHR 1 | MP5 | Dec-10 | Johor Bharu,Johor | N 01° 27.705 | E 103° 45.253 | x | x | x |
|  | **SINGAPORE [*n* = 9, *Ne* = 8, H = 5, H­­_d_ = 0.63]** | | | | | | | | |
| 17 | ST1 | SG1 | Mar-10 | W50 S20 South, Keppel Road,Singapore | N 01° 16.411 | E 103° 50.260 | x | x | x |
| 18 | ET1 | MP1/SG | Mar-10 | W50 S20 East, Upper Changi Road,Singapore | N 01° 20.079 | E 103° 57.060 | x | x | x |
| 19 | NT1 | SG2 | Mar-10 | W50 S20 North, Yio Chu Kang, Singapore | N 01° 22.898 | E 103° 50.672 | x | x | x |
| 20 | WT1 | SG3 | Mar-10 | W50 S20 West, Yunnan Crescent,Jurong west, Singapore | N 01° 20.325 | E 103° 41.529 | x | x | x |
| 21 | SG1 | SG4 | Mar-10 | 2 Cornwall road, Singapore | N 01° 16.893 | E 103° 48.000 | x | x | x |
| 22 | SG2 | MP1/SG | Mar-10 | Vista Exchange green (behind Rochester Park) Singapore | N 01° 18.362 | E 103° 47.289 | x | x | x |
| 23 | SG3 | MP1/SG | Mar-10 | Outside Thomson Grove Condo,Singapore | N 01° 23.408 | E 103° 49.915 | x | x | x |
| 24 | SG4 | - | Mar-10 | Yio Chu Kang Road (B50),Singapore | N 01° 22.812 | E 103° 50.657 | x |  |  |
| 25 | SG5 | MP1/SG | Mar-10 | Opposite 2 Adam Road (vacant land), Singapore | N 01° 19.764 | E 103° 48.880 | x | x | x |
|  | **THE PHILIPPINES [*n* = 6, *Ne* = 6, H = 4, H_d_ = 0.67]** | | | | | | | | |
| 26 | ALM | PP1 | May-10 | Alaminos, the Phillipines | N 16° 09.658 | E 119° 58.828 | x | x | x |
| 27 | CAL | PP1 | May-10 | Calamba, the Phillipines | N 14° 11.340 | E 121° 07.379 | x | x | x |
| 28 | CAV | PP1 | May-10 | Cavite, the Phillipines | N 14° 28.983 | E 120° 53.791 | x | x | x |
| 29 | DAV | PP2 | May-10 | Davao, the Phillipines | N 07° 11.478 | E 125° 27.395 | x | x | x |
| 30 | CEB | PP3 | May-10 | Cebu, the Phillipines | N 10° 19.003 | E 123° 52.923 | x | x | x |
| 31 | MAN 1 | PP4 | May-10 | Manila, the Phillipines | N 14° 36.594 | E 120° 59.280 | x | x | x |
|  | **BORNEO [*n* = 28, *Ne* = 26, H = 7, H_d_ = 0.27]** | | | | | | | | |
| 32 | LA | BN1 | Aug-10 | Lubok Antu, Sarawak | N 01° 05.690 | E 111° 49.191 | x | x | x |
| 33 | BTG | BN2 | Aug-10 | Bintangor, Sarawak | N 02° 09.977 | E 111° 38.390 | x | x | x |
| 34 | DAM | BN2 | Aug-10 | Damai, Sarawak | N 01° 44.311 | E 110° 18.995 | x | x | x |
| 35 | ASJ 20Q | BN2 | Aug-10 | Asajaya, Sarawak | N 01° 32.518 | E 110° 36.844 | x | x | x |
| 36 | SRK 66Q | BN2 | Aug-10 | Sarikei, Sarawak | N 02° 07.715 | E 111° 31.379 | x | x | x |
| 37 | RI | BN2 | Aug-10 | Balai Ringin, Sarawak | N 01° 02.978 | E 110° 45.379 | x | x | x |
| 38 | MT 11Q | BN2 | Aug-10 | Matang, Sarawak | N 01° 37.018 | E 110° 09.421 | x | x | x |
| 39 | BAU 23Q | BN2 | Aug-10 | Bau, Sarawak | N 01° 24.915 | E 110° 09.102 | x | x | x |
| 40 | END 14Q | BN2 | Aug-10 | Endap, Sarawak | N 01° 23.770 | E 110°27.295 | x | x | x |
| 41 | LD 27Q | BN2 | Aug-10 | Lundu, Sarawak | N 01° 38.829 | E 109° 50.744 | x | x | x |
| 42 | BLU 51Q | BN2 | Aug-10 | Batang Lupar, Sarawak | N 01° 09.092 | E 111° 38.947 | x | x | x |
| 43 | TEB 34Q | BN2 | Aug-10 | Tebakang/Serian, Sarawak | N 01° 08.747 | E 110° 32.196 | x | x | x |
| 44 | SIB | BN3 | Aug-10 | Sibu, Sarawak | N 02° 13.895 | E 111° 49.191 | x | x | x |
| 45 | WSE | - | Jan-11 | Wawasan Sedar Sdn. Bhd.,Bintulu, Sarawak | N 03° 11.306 | E 113° 05.364 |  | x |  |
| 46 | WB1 | BN4 | Jan-11 | Wawasan Sedar Sdn. Bhd.,Bintulu, Sarawak | N 03° 11.281 | E 113° 05.339 | x | x | x |
| 47 | WB2 | BN4 | Jan-11 | Wawasan Sedar Sdn. Bhd.,Bintulu, Sarawak | N 03° 11.320 | E 113° 05.369 | x | x | x |
| 48 | WB4 | BN4 | Jan-11 | Wawasan Sedar Sdn. Bhd.,Bintulu, Sarawak | N 03° 11.314 | E 113° 05.304 | x | x | x |
| 49 | WB3 | BN5 | Jan-11 | Wawasan Sedar Sdn. Bhd.,Bintulu, Sarawak | N 03° 11.330 | E 113° 05.385 | x | x | x |
| 50 | MR2 | BN5 | Apr-11 | Miri, Sarawak | N 04° 21.908 | E 114° 01.347 | x | x | x |
| 51 | MR3 | BN5 | Apr-11 | Miri, Sarawak | N 04° 22.035 | E 114° 01.480 | x | x | x |
| 52 | MR4 | BN5 | Apr-11 | Miri, Sarawak | N 04° 22.193 | E 114° 01.471 | x | x | x |
| 53 | MR5 | BN5 | Apr-11 | Miri, Sarawak | N 04° 21.701 | E 114° 01.550 | x | x | x |
| 54 | MR1 | BN6 | Apr-11 | Miri, Sarawak | N 04° 21.859 | E 114° 01.407 | x | x | x |
| 55 | MGT1 | - | Feb-11 | Menggatal,Kota Kinabalu, Sabah | N 06° 01.366 | E 116° 09.404 |  | x |  |
| 56 | MGT2 | BN5 | Feb-11 | Menggatal,Kota Kinabalu, Sabah | N 06° 01.353 | E 116° 09.401 | x | x | x |
| 57 | INAM | BN5 | Feb-11 | Inanam, Sabah | N 05° 59.559 | E 116° 07.954 | x | x | x |
| 58 | SMR1 | BN7 | Jan-11 | Samarinda, East Kalimantan, Indonesia | S 00° 30.811 | E 117° 09.521 | x | x | x |
| 59 | SMR3 | BN7 | Jan-11 | Samarinda, East Kalimantan, Indonesia | S 00° 30.799 | E 117° 09.500 | x | x | x |
|  | **THAILAND [*n* = 10, *Ne* = 5, H = 4, H_d_ = 0.80]** | | | | | | | | |
| 60 | THAI2 | TH1 | Oct-10 | Bangkok, Thailand | N 13° 43.505 | E 100° 28.507 | x | x | x |
| 61 | THAI3 | TH1 | Oct-10 | Bangkok, Thailand | N 13° 43.493 | E 100° 28.512 | x | x | x |
| 62 | THA | TH2 | Apr-11 | Chainat,North Thailand | N 15° 05.597 | E 099° 59.437 | x | x | x |
| 63 | THB | TH3 | Apr-11 | Chainat,North Thailand | N 15° 05.614 | E 099° 59.404 | x | x | x |
| 64 | THD | - | Apr-11 | Phichit,North Thailand | N 16° 11.287 | E 100° 20.989 |  |  |  |
| 65 | THE | - | Apr-11 | Phichit,North Thailand | N 16° 11.314 | E 100° 20.991 | x |  |  |
| 66 | THG | TH4 | Apr-11 | Ratchatani,North Thailand | N 15° 04.367 | E 105° 13.120 | x | x | x |
| 67 | THH | - | Apr-11 | Nakhonsawan,North Thailand | N 15° 44.042 | E 100° 24.200 |  | x |  |
| 68 | THI | - | Apr-11 | Nakhonsawan,North Thailand | N 15° 43.988 | E 100° 24.144 |  | x |  |
| 69 | THJ | - | Apr-11 | Nakhonsawan,North Thailand | N 15° 43.919 | E 100° 24.017 |  | x |  |
|  | **VIETNAM [*n* = 10, *Ne* = 6, H = 5, H_d_ = 0.83]** | | | | | | | | |
| 70 | VIET | - | Jan-11 | Bien Hoa city,Vietnam | N 10° 56.461 | E 106° 52.913 |  | x |  |
| 71 | V1 | VT1 | Dec-09 | Dak lak, (Krong Pak Thuong), Vietnam | N 12° 39.777 | E 108° 08.374 | x | x | x |
| 72 | V9 | VT1 | Sep-10 | Dak lak (Eabong dam) Vietnam | N 12° 32.063 | E 108° 03.536 | x | x | x |
| 73 | V2 | - | Jun-08 | Dak Nong,(Tuy Duc),Vietnam | N 12° 08.988 | E 107° 23.148 |  | x |  |
| 74 | V6 | - | Sep-10 | Ninh Thuan (Ocam damp) Vietnam | N 11° 38.533 | E 108° 53.523 |  | x |  |
| 75 | V4 | VT2 | Jun-10 | Phu Yen (Ky Chau)Vietnam | N 13° 16.897 | E 109° 04.469 | x | x | x |
| 76 | V5 | VT3 | Nov-09 | Lam Dong (Cai Baoy)Vietnam | N 11° 18.464 | E 108° 08.286 | x | x | x |
| 77 | V8 | VT4 | Jun-10 | Gia Lai (Iamo dam),Vietnam | N 13° 48.801 | E 108° 05.619 | x | x | x |
| 78 | V7 | VT5 | Apr-10 | Dak lak (Hoa Thang), Vietnam | N 12° 39.481 | E 108° 06.779 | x | x | x |
| 79 | V10 |  | Apr-05 | Phu Yen (Baha dam)Vietnam | N 13° 16.081 | E 109° 08.710 | x |  |  |
|  | **JAVA [*n* = 33, *Ne* = 28, H = 13, H_d_ = 0.46]** | | | | | | | | |
| 80 | CI1 | JV1 | Dec-11 | Cibinong, Jakarta, West Java, Indonesia | S 06° 28.571 | E 106° 51.128 | x | x | x |
| 81 | SR1 | JV1 | Dec-11 | Serpong, Jakarta, West Java, Indonesia | S 06° 19.148 | E 106° 40.822 | x | x | x |
| 82 | BAND | JV2 | Dec-11 | Bandung, West Java, Indonesia | S 06° 54.786 | E 107° 36.550 | x | x | x |
| 83 | Y1 | JV3 | Oct-11 | Magelang, Sala Tiga, Kopeng, G. Merbabu, Central Java | S 07° 28.931 | E 110° 12.832 | x | x | x |
| 84 | Y10 | JV4 | Oct-11 | Depok,Sleman, Central Java | S 07° 43.856 | E 110° 24.012 | x | x | x |
| 85 | Y14 | JV5 | Oct-11 | Hutan Wanagama (UGM) Central Java | S 08° 01.873 | E 110° 36.602 | x | x | x |
| 86 | Y15 | JV6 | Oct-11 | Hutan Wanagama (UGM) Central Java | S 08° 01.777 | E 110° 35.557 | x | x | x |
| 87 | Y17 | JV7 | Oct-11 | Forest institute, closer to Wonosari, Central Java | S 07° 58.295 | E 110° 35.832 | x | x | x |
| 88 | Y18 | JV5 | Oct-11 | Forest institute, closer to Wonosari, Central Java | S 07° 58.778 | E 110° 33.565 | x | x | x |
| 89 | Y19 | JV8 | Oct-11 | Bantul, Central Java | S 07° 55.140 | E 110° 22.708 | x | x | x |
| 90 | Y20 | JV9 | Oct-11 | Bantul, Central Java | S 07° 53.145 | E 110° 23.657 | x | x | x |
| 91 | Y21 | JV4 | Oct-11 | Bantul, Central Java | S 07° 54.235 | E 110° 25.678 | x | x | x |
| 92 | Y22 | JV3 | Oct-11 | Bantul, Central Java | S 07° 53.223 | E 110° 24.012 | x | x | x |
| 93 | M4 | - | Oct-11 | Kampung Murakung, Bangkalan, Madura | S 07° 03.772 | E 112° 46.698 |  | x |  |
| 94 | M6 | JV10 | Oct-11 | Kampung Murakung, Bangkalan, Madura | S 07° 03.740 | E 112° 46.749 | x | x | x |
| 95 | M8 | JV11 | Oct-11 | Kampung Murakung, Bangkalan, Madura | S 07° 03.671 | E 112° 46.680 | x | x | x |
| 96 | M9 | - | Oct-11 | Kampung Murakung, Bangkalan, Madura | S 07° 03.719 | E 112° 46.654 | x |  |  |
| 97 | M12 | JV11 | Oct-11 | Kampung Murakung, Bangkalan, Madura | S 07° 03.747 | E 112° 46.645 | x | x | x |
| 98 | M18 | JV11 | Oct-11 | Kampung Murakung, Bangkalan, Madura | S 07° 03.793 | E 112° 46.543 | x | x | x |
| 99 | M20 | JV11 | Oct-11 | Kampung Murakung, Bangkalan, Madura | S 07° 03.774 | E 112° 46.677 | x | x | x |
| 100 | M1 | - | Oct-11 | Kampung Murakung, Bangkalan, Madura | S 07° 03.792 | E 112° 46.733 | x |  |  |
| 101 | M3 | JV11 | Oct-11 | Kampung Murakung, Bangkalan, Madura | S 07° 03.834 | E 112° 46.784 | x | x | x |
| 102 | M11 | JV12 | Oct-11 | Kampung Murakung, Bangkalan, Madura | S 07° 03.652 | E 112° 46.702 | x | x | x |
| 103 | K4 | JV8 | Oct-11 | Desa Bareng Krajan,Krian,Sidoarjo, East Java | S 07° 25.347 | E 112° 40.983 | x | x | x |
| 104 | K1 | JV11 | Oct-11 | Desa Bareng Krajan,Krian,Sidoarjo, East Java | S 07° 25.396 | E 112° 41.164 | x | x | x |
| 105 | K2 | JV11 | Oct-11 | Desa Bareng Krajan,Krian,Sidoarjo, East Java | S 07° 25.394 | E 112° 41.148 | x | x | x |
| 106 | K3 | JV13 | Oct-11 | Desa Bareng Krajan,Krian,Sidoarjo, East Java | S 07° 25.393 | E 112° 41.129 | x | x | x |
| 107 | K5a | - | Oct-11 | Desa Bareng Krajan,Krian,Sidoarjo, East Java | S 07° 25.431 | E 112° 41.213 | x |  |  |
| 108 | K6 | JV11 | Oct-11 | Desa Bareng Krajan,Krian,Sidoarjo, East Java | S 07° 25.476 | E 112° 41.242 | x | x | x |
| 109 | S1 | - | Oct-11 | Institut Teknologi Sepuluh Nopember, Surabaya, East Java | S 07° 16.996 | E 112° 47.697 | x |  |  |
| 110 | S2 | JV11 | Oct-11 | Institut Teknologi Sepuluh Nopember, Surabaya, East Java | S 07° 16.966 | E 112° 47.703 | x | x | x |
| 111 | S3 | JV11 | Oct-11 | Institut Teknologi Sepuluh Nopember, Surabaya, East Java | S 07° 16.998 | E 112° 47,716 | x | x | x |
| 112 | S4 | JV11 | Oct-11 | Institut Teknologi Sepuluh Nopember, Surabaya, East Java | S 07° 16.968 | E 112° 47.723 | x | x | x |
|  | **SUMATRA [*n* = 48, *Ne* = 48, H = 9, H_d_ = 0.19]** | | | | | | | | |
| 113 | SU1 | MP3/SU | Jan-12 | Secanggang, Langkat, North Sumatra (oil palm estate) | N 03° 52.241 | E 098° 32.081 | x | x | x |
| 114 | SU6 | MP3/SU | Jan-12 | Secanggang, Langkat, North Sumatra (oil palm estate) | N 03° 52.311 | E 098° 32.338 | x | x | x |
| 115 | SU8 | MP3/SU | Jan-12 | Secanggang, Langkat, North Sumatra (oil palm estate) | N 03° 52.294 | E 098° 32.351 | x | x | x |
| 116 | SU11 | MP3/SU | Jan-12 | Secanggang, Langkat, North Sumatra (oil palm estate) | N 03° 52.339 | E 098° 32.362 | x | x | x |
| 117 | SU13 | MP3/SU | Jan-12 | Secanggang, Langkat, North Sumatra (oil palm estate) | N 03° 52.328 | E 098° 32.389 | x | x | x |
| 118 | SU2 | SU1 | Jan-12 | Secanggang, Langkat, North Sumatra (oil palm estate) | N 03° 52.269 | E 098° 32.322 | x | x | x |
| 119 | SU4 | SU1 | Jan-12 | Secanggang, Langkat, North Sumatra (oil palm estate) | N 03° 52.291 | E 098° 32. 328 | x | x | x |
| 120 | SU7 | SU1 | Jan-12 | Secanggang, Langkat, North Sumatra (oil palm estate) | N 03° 52.308 | E 098° 32.352 | x | x | x |
| 121 | SU9 | SU1 | Jan-12 | Secanggang, Langkat, North Sumatra (oil palm estate) | N 03° 52.276 | E 098° 32.338 | x | x | x |
| 122 | SU14 | SU1 | Jan-12 | Secanggang, Langkat, North Sumatra (oil palm estate) | N 03° 52.330 | E 098° 32.398 | x | x | x |
| 123 | SU15 | SU1 | Jan-12 | Secanggang, Langkat, North Sumatra (oil palm estate) | N 03° 52.333 | E 098° 32.387 | x | x | x |
| 124 | SU10 | SU8 | Jan-12 | Secanggang, Langkat, North Sumatra (oil palm estate) | N 03° 52.284 | E 098° 32.334 | x | x | x |
| 125 | SU3 | SU2 | Jan-12 | Secanggang, Langkat, North Sumatra (oil palm estate) | N 03° 52.266 | E 098° 32.324 | x | x | x |
| 126 | SU12 | SU2 | Jan-12 | Secanggang, Langkat, North Sumatra (oil palm estate) | N 03° 52.331 | E 098° 32.366 | x | x | x |
| 127 | SU5 | SU3 | Jan-12 | Secanggang, Langkat, North Sumatra (oil palm estate) | N 03° 52.313 | E 098° 32.332 | x | x | x |
| 128 | SU16 | MP3/SU | Jan-12 | Binjai, North Sumatra (oil palm estate) | N 03° 39.014 | E 098° 30.987 | x | x | x |
| 129 | SU18 | MP3/SU | Jan-12 | Binjai, North Sumatra (oil palm estate) | N 03° 39.009 | E 098° 30.995 | x | x | x |
| 130 | SU19 | MP3/SU | Jan-12 | Binjai, North Sumatra (oil palm estate) | N 03° 38.994 | E 098° 30.883 | x | x | x |
| 131 | SU20 | MP3/SU | Jan-12 | Binjai, North Sumatra (oil palm estate) | N 03° 38.992 | E 098° 30.886 | x | x | x |
| 132 | SU17 | SU1 | Jan-12 | Binjai, North Sumatra (oil palm estate) | N 03° 39.014 | E 098° 30.977 | x | x | x |
| 133 | SU21 | SU1 | Jan-12 | Binjai, North Sumatra (oil palm estate) | N 03° 39.006 | E 098° 30.867 | x | x | x |
| 134 | R1 | SU4 | Jan-12 | Universitas Riau (oil palm trees), Riau | N 00° 28.816 | E 101° 22.665 | x | x | x |
| 135 | R2 | SU4 | Jan-12 | Universitas Riau (oil palm trees), Riau | N 00° 28.881 | E 101° 22.662 | x | x | x |
| 136 | R3 | SU4 | Jan-12 | Universitas Riau (oil palm trees), Riau | N 00° 28. 883 | E 101° 22.669 | x | x | x |
| 137 | R7 | SU4 | Jan-12 | Rimbo Panjang, Tambang, Kampar Riau (rubber estate) | N 00° 25.424 | E 101° 17.743 | x | x | x |
| 138 | R8 | SU4 | Jan-12 | Rimbo Panjang, Tambang, Kampar Riau (rubber estate) | N 00° 25. 390 | E 101° 17.769 | x | x | x |
| 139 | R9 | SU4 | Jan-12 | Rimbo Panjang, Tambang, Kampar Riau (rubber estate) | N 00° 25.351 | E 101° 17.771 | x | x | x |
| 140 | R6 | SU5 | Jan-12 | Rimbo Panjang, Tambang, Kampar Riau (rubber estate) | N 00° 25.441 | E 101° 17.724 | x | x | x |
| 141 | R11 | SU4 | Jan-12 | Desa Indrapura, Rombio Jaya,Kampar Riau (oil palm estate) | N 00° 26.858 | E 101° 10.550 | x | x | x |
| 142 | R12 | SU4 | Jan-12 | Desa Indrapura, Rombio Jaya,Kampar Riau (oil palm estate) | N 00° 26.878 | E 101° 10.572 | x | x | x |
| 143 | R13 | SU4 | Jan-12 | Desa Indrapura, Rombio Jaya,Kampar Riau (oil palm estate) | N 00° 26.882 | E 101° 10.584 | x | x | x |
| 144 | R14 | SU4 | Jan-12 | Desa Indrapura, Rombio Jaya,Kampar Riau (oil palm estate) | N 00° 26.898 | E 101° 10.587 | x | x | x |
| 145 | R15 | SU4 | Jan-12 | Desa Indrapura, Rombio Jaya,Kampar Riau (oil palm estate) | N 00° 26.948 | E 101° 10.578 | x | x | x |
| 146 | R19 | SU4 | Jan-12 | Desa Indrapura, Rombio Jaya,Kampar Riau (oil palm estate) | N 00° 26.747 | E 101° 10.479 | x | x | x |
| 147 | R18 | SU6 | Jan-12 | Desa Indrapura, Rombio Jaya,Kampar Riau (oil palm estate) | N 00° 26.736 | E 101° 10.493 | x | x | x |
| 148 | P2 | SU4 | Jan-12 | Lubok Basung, Agam (oil palm estate), West Sumatra | S 00° 15.732 | E 099° 59.178 | x | x | x |
| 149 | P3 | SU4 | Jan-12 | Lubok Basung, Agam (oil palm estate), West Sumatra | S 00° 15.792 | E 099° 59.183 | x | x | x |
| 150 | P4 | SU4 | Jan-12 | Lubok Basung, Agam (oil palm estate), West Sumatra | S 00° 15.829 | E 099° 59.201 | x | x | x |
| 151 | P5 | SU4 | Jan-12 | Lubok Basung, Agam (oil palm estate), West Sumatra | S 00° 15.833 | E 099° 59.203 | x | x | x |
| 152 | P6 | SU4 | Jan-12 | Lubok Basung, Agam (oil palm estate), West Sumatra | S 00° 15.817 | E 099° 59.110 | x | x | x |
| 153 | P7 | SU4 | Jan-12 | Lubok Basung, Agam (oil palm estate), West Sumatra | S 00° 15.780 | E 099° 59.077 | x | x | x |
| 154 | P9 | SU4 | Jan-12 | Lubok Basung, Agam (oil palm estate), West Sumatra | S 00° 15.768 | E 099° 59.132 | x | x | x |
| 155 | P10 | SU4 | Jan-12 | Lubok Basung, Agam (oil palm estate), West Sumatra | S 00° 15.745 | E 099° 59.089 | x | x | x |
| 156 | P12 | SU4 | Jan-12 | Lintau Buo, Tanah Datar, Pangian (paddy field), W. Sumatra | S 00° 28.132 | E 100° 46.105 | x | x | x |
| 157 | P13 | SU4 | Jan-12 | Lintau Buo, Tanah Datar, Pangian (paddy field), W. Sumatra | S 00° 28.129 | E 100° 46.094 | x | x | x |
| 158 | 1koto | SU4 | Sep-10 | Koto, Sumatera, Indonesia, West Sumatra | S 00° 28.122 | E 099° 46.132 | x | x | x |
| 159 | 2koto | SU4 | Sep-10 | Koto, Sumatera, Indonesia, West Sumatra | S 00° 28.111 | E 099° 46.332 | x | x | x |
| 160 | 1padang | SU7 | Sep-10 | Padang, Sumatera, Indonesia, West Sumatra | S 00° 28.723 | E 100° 48.112 | x | x | x |

*n* = total sample size; *Ne* = effective sample size used in combined dataset; H = total haplotypes; H_d_ = Haplotype diversity.

* Samples were collected from termite nests located at open parks, underneath the trees along roadsides and urban landscapes unless otherwise mentioned.
