# Supplementary material for: Phylogeography of the termite Macrotermes gilvus and insight into ancient dispersal corridors in Pleistocene Southeast Asia
Source: PLoS One. 2017 Nov 29;12(11):e0186690. doi: 10.1371/journal.pone.0186690 (PMC5706666; doi:10.1371/journal.pone.0186690)
Supplement: S2 Table — (DOCX) [file pone.0186690.s002.docx]

**S2 Table. Additional *M. gilvus* samples used in microsatellite analysis.**

| **No** | **Code** | **Date** | **Localities** | **Latitude** | **Longitude** |
| --- | --- | --- | --- | --- | --- |
| 161 | 003P | Nov-10 | Lenggong, Perak | N 05° 07.600 | E 100° 59.599 |
| 162 | SDG2 | Nov-10 | Serdang, Selangor | N 02° 59.533 | E 101° 42.577 |
| 163 | SDG3 | Nov-10 | Serdang, Selangor | N 02° 58.761 | E 101° 41.595 |
| 164 | 3K | Jul-10 | Alor setar | N 06° 07.165 | E 100° 22.032 |
| 165 | TRM2 | Jul-10 | Teluk Bahang, Penang | N 05° 26.812 | E 100° 12.974 |
| 166 | BP1 | Jul-10 | Balik Pulau,Penang | N 05° 22.552 | E 100° 12.778 |
| 167 | BP2 | Dec-10 | Balik Pulau,Penang | N 05° 22.611 | E 100° 12.812 |
| 168 | JHR2 | Dec-10 | Johor Bharu, Johor | N 01° 27.711 | E 103° 45.223 |
| 169 | JHR3 | Dec-10 | Johor Bharu, Johor | N 01° 27.700 | E 103° 45.103 |
| 170 | JHR4 | Dec-10 | Johor Bharu, Johor | N 01° 27.623 | E 103° 45.357 |
| 171 | JHR5 | Dec-10 | Johor Bharu, Johor | N 01° 27.573 | E 103° 45.121 |
| 172 | Aitam | Sep-11 | Air Itam, Penang | N 05° 23.993 | E 100° 17.176 |
| 173 | Aitam3 | Sep-11 | Air Itam, Penang | N 05° 24.033 | E 100° 17.128 |
| 174 | Mhead | Sep-11 | Mukahead, Penang | N 05° 27.650 | E 100° 12.329 |
| 175 | Tbungah | Sep-11 | Tanjung Bungah, Penang | N 05° 27.861 | E 100° 17.806 |
| 176 | SDG4 | Jul-10 | Serdang, Selangor | N 02° 59.500 | E 101° 41.628 |
| 177 | SDG5 | Jul-10 | Serdang, Selangor | N 02° 59.311 | E 101° 41.722 |
| 178 | 001P | Jul-10 | Lenggong, Perak | N 05° 07.501 | E 100° 59.391 |
| 179 | Aitam2 | Jul-10 | Air Itam, Penang | N 05° 23.711 | E 100° 17.200 |
| 180 | GG4 | Jul-10 | Gelugor, Penang | N 05° 21.687 | E 100° 18.209 |
| 181 | NTH | Mar-10 | Yio Chu Kang, Singapore | N 01° 22.788 | E 103° 50.709 |
| 182 | Thai1 | Oct-10 | Bangkok, Thailand | N 13° 43.483 | E 100° 28.522 |
| 183 | THC | Oct-10 | Thailand | N 13° 43.470 | E 100° 28.522 |
| 184 | THF | Oct-10 | Thailand | N 13° 43.350 | E 100° 28.522 |
| 185 | R4 | Jan-12 | Riau, Sumatra | N 00° 28.879 | E 101° 22.670 |
| 186 | R5 | Jan-12 | Riau, Sumatra | N 00° 28.880 | E 101° 22.551 |
| 187 | R10 | Jan-12 | Riau, Sumatra | N 00° 25.440 | E 101° 17.711 |
| 188 | R16 | Jan-12 | Riau, Sumatra | N 00° 26.811 | E 101° 10.491 |
| 189 | R17 | Jan-12 | Riau, Sumatra | N 00° 26.830 | E 101° 10.477 |
| 190 | DAMAI | Aug-10 | Damai, Sarawak | N 01° 38.450 | E 110° 20.482 |
| 191 | 038Q | Aug-10 | Balai Ringin, Sarawak | N 01° 02.928 | E 110° 45.470 |
| 192 | 058Q | Aug-10 | Kanowit, Sarawak | N 02° 04.729 | E 112° 01.476 |
| 193 | 040Q | Aug-10 | Betong, Sarawak | N 01° 24.423 | E 111° 31.555 |
| 194 | 041Q | Aug-10 | Betong, Sarawak | N 01° 24.377 | E 111° 31.431 |
| 195 | 043Q | Aug-10 | Betong, Sarawak | N 01° 24.494 | E 111° 31.679 |
| 196 | 054Q | Aug-10 | Sibu, Sarawak | N 02° 13.900 | E 111° 49.211 |
| 197 | 056Q | Aug-10 | Sibu, Sarawak | N 02° 13.976 | E 111° 49.262 |
| 198 | 047Q | Aug-10 | Debak, Sarawak | N 01° 33.772 | E 111° 25.338 |
| 199 | 053Q | Aug-10 | Sri Aman, Sarawak | N 01° 13.160 | E 111° 28.025 |
| 200 | 057Q | Aug-10 | Kanowit, Sarawak | N 02° 04.533 | E 112° 01.800 |
| 201 | 060Q | Aug-10 | Kanowit, Sarawak | N 02° 04.601 | E 112° 01.879 |
| 202 | 050Q | Aug-10 | Lubok Antu, Sarawak | N 01° 05.757 | E 111° 49.149 |
| 203 | SRKI | Aug-10 | Sarikei. Sarawak | N 02° 07.711 | E 111° 31.371 |
| 204 | Y2 | Oct-11 | Yogjakarta,Central Java | S 07° 55.140 | E 110° 22.708 |
| 205 | Y4 | Oct-11 | Yogjakarta,Central Java | S 07° 53.145 | E 110° 23.657 |
| 206 | Y11 | Oct-11 | Yogjakarta,Central Java | S 08° 01.846 | E 110° 36.611 |
| 207 | Y13 | Oct-11 | Yogjakarta,Central Java | S 08° 01.800 | E 110° 36.500 |
| 208 | M19 | Oct-11 | Bangkalan, Madura | S 07° 03.700 | E 112° 46.677 |
| 209 | M17 | Oct-11 | Bangkalan, Madura | S 07° 03.612 | E 112° 46.587 |
| 210 | M10 | Oct-11 | Bangkalan, Madura | S 07° 03.555 | E 112° 46.601 |
| 211 | M14 | Oct-11 | Bangkalan, Madura | S 07° 03.631 | E 112° 46.423 |
| 212 | M16 | Oct-11 | Bangkalan, Madura | S 07° 03.778 | E 112° 46.557 |
| 213 | M15 | Oct-11 | Bangkalan, Madura | S 07° 03.872 | E 112° 46.302 |
