# Supplementary material for: Phylogeography of the termite Macrotermes gilvus and insight into ancient dispersal corridors in Pleistocene Southeast Asia
Source: PLoS One. 2017 Nov 29;12(11):e0186690. doi: 10.1371/journal.pone.0186690 (PMC5706666; doi:10.1371/journal.pone.0186690)
Supplement: S3 Table — (DOCX) [file pone.0186690.s003.docx]

**S3 Table. Best-fit nucleotide substitution model based on hierarchical likelihood ratio test (hLRT) and Akaike’s information criteria (AIC).**

| **Subsets** | **hLRT** | **AIC** |
| --- | --- | --- |
| All genes combined | GTR+I+G **(-lnL 3959.8286)** | GTR+I+G **(-lnL 3959.8286)** |
| Noncoding (16S rRNA) | TrN+G **(-lnL 1343.2306)** | GTR+I**(-lnL 1335.7816)** |
| Coding (COII) | TrN+I+G **(-lnL 2519.3948)** | TrN+I+G **(-lnL 2519.39480)** |
| 1stcodon | TrN+G **(-lnL 594.4199)** | TrN+I **(-lnL 594.2953)** |
| 2ndcodon | F81 **(-lnL 413.6479)** | TrN+I **(-lnL 410.5594)** |
| 3rdcodon | HKY+G **(-lnL 1244.6324)** | GTR+G **(-lnL 1240.0416)** |
| 1stcocon2ndcodon | HKY+G | TVM + I |
