# Supplementary material for: Phylogeography of the termite Macrotermes gilvus and insight into ancient dispersal corridors in Pleistocene Southeast Asia
Source: PLoS One. 2017 Nov 29;12(11):e0186690. doi: 10.1371/journal.pone.0186690 (PMC5706666; doi:10.1371/journal.pone.0186690)
Supplement: S4 Table — Numbers in bracket represents 1st, 2nd and 3rd codon positions of COII gene respectively. Partitioning scheme with best AIC score is highlighted in bold. (DOCX) [file pone.0186690.s004.docx]

**S4 Table. Partitioning schemes for the concatenated dataset based on GARLI and MrBayes optimization process.** Numbers in bracket represents 1^st^, 2^nd^ and 3^rd^ codon positions of COII gene respectively. Partitioning scheme with best AIC score is highlighted in bold.

| **Partition Schemes** | **Models** | **GARLI** | | | **MrBayes** |
| --- | --- | --- | --- | --- | --- |
|  |  | **Likelihood scores (lnL)** | **Total parameters** | **AIC scores** | **Harmonic means** |
| (123)noncoding | TrN+I+G; GTR+I | -3932.9839 | 20 | 7905.968 | -4216.54 |
| (12)(3)noncoding | TVM+I; GTR+G; GTR+I | -3777.8638 | 28 | 7611.728 | -4092.15 |
| All genes combined | GTR+I+G | -3953.0745 | 10 | 7926.149 | -4110.44 |
| (1)(2)(3)noncoding | TrN+I; TrN+I; GTR+G; GTR+I | -3749.1664 | 39 | **7576.333** | **-**4055.59 |
