# Supplementary material for: Phylogeography of the termite Macrotermes gilvus and insight into ancient dispersal corridors in Pleistocene Southeast Asia
Source: PLoS One. 2017 Nov 29;12(11):e0186690. doi: 10.1371/journal.pone.0186690 (PMC5706666; doi:10.1371/journal.pone.0186690)
Supplement: S5 Table — Best partitioning scheme for Bayesian inference is shown in bold. Italicized values indicate number of parameters used to calculate the bayes factor. (DOCX) [file pone.0186690.s005.docx]

**S5 Table. Bayes factor analysis.** Best partitioning scheme for bayesian inference is shown in bold. Italicized values indicate number of parameters used to calculate the bayes factor.

| **Partition schemes**  **(2ln Bayes factor)** | **(123)noncoding**  ***(19)*** | **(12)(3)noncoding**  ***(26)*** | **single concatenated**  **(*10*)** | **(1)(2)(3)noncoding**  ***(36)*** |
| --- | --- | --- | --- | --- |
| (123)noncoding | * |  |  |  |
| (12)(3)noncoding | 9.647 | * |  |  |
| single concatenated | 9.329 | -5.813 | * |  |
| **(1)(2)(3)noncoding** | 10.162 | 7.198 | 8.009 | * |
|  |  |  |  |  |
| **Interpretation of the Bayes factor (B10) taken from Kass and Raftery [85]** | | | | |
| 2ln (B10) | B10 | Evidence against Mo |  |  |
| 0 to 2 | 1 to 3 | not worth more than a bare mention |  |  |
| 2 to 6 | 3 to 20 | positive |  |  |
| 6 to 10 | 20 to 150 | strong |  |  |
| > 10 | > 150 | very strong |  |  |

Reference:

85. Kass RE, Raftery AE. Bayes factors. J Am Stat Assoc. 1995; 90: 773–795.
