# Supplementary material for: Phylogeography of the termite Macrotermes gilvus and insight into ancient dispersal corridors in Pleistocene Southeast Asia
Source: PLoS One. 2017 Nov 29;12(11):e0186690. doi: 10.1371/journal.pone.0186690 (PMC5706666; doi:10.1371/journal.pone.0186690)
Supplement: S6 Table — (DOCX) [file pone.0186690.s006.docx]

**S6 Table. Maximum likelihood values and statistics for the alternative phylogenetic hypotheses calculated in GARLI and CONSEL.**

| **Hypothesis** | **Likelihood scores (lnL)** | **AU** | **KH** | **SH** | **WKH** | **WSH** |
| --- | --- | --- | --- | --- | --- | --- |
| This study | - 3749.171 | 0.703 | 0.71 | 0.949 | 0.71 | 0.958 |
| Vietnam monophyly | - 3844.787 | 0.297 | 0.29 | 0.559 | 0.29 | 0.486 |
| Sumatra monophyly | - 3796.949 | <0.001* | <0.001* | <0.001* | <0.001* | <0.001* |
| Borneo monophyly | - 3755.026 | <0.001* | <0.001* | <0.001* | <0.001* | <0.001* |

*Statistically significant at *P* < 0.05 rejecting the hypothesis tested.
