# Supplementary material for: Phylogeography of the termite Macrotermes gilvus and insight into ancient dispersal corridors in Pleistocene Southeast Asia
Source: PLoS One. 2017 Nov 29;12(11):e0186690. doi: 10.1371/journal.pone.0186690 (PMC5706666; doi:10.1371/journal.pone.0186690)
Supplement: S7 Table — (DOCX) [file pone.0186690.s007.docx]

**S7 Table. Pairwise Φ_ST_ values (lower diagonal) and geographical Euclidean distance (shortest distance between pairs of populations, km) used in isolation-by-distance analysis (upper diagonal).**

|  | Mainland Southeast Asia | | | | Sumatra | | | Borneo | | Philippines | Java | | |
| --- | --- | --- | --- | --- | --- | --- | --- | --- | --- | --- | --- | --- | --- |
|  | TH | VT | MP | SG | NS | RI | WS | B1 | B2 | PP | CJ | EJ | MD |
| TH | * | 913.2 | 1222.2 | 1545.7 | 1292.5 | 1612.2 | 1684.6 | 1889.1 | 1979.0 | 2591.2 | 2708.8 | 2793.6 | 2767.3 |
| VT | **0.47** | * | 1143.8 | 1318.5 | 1503.4 | 1528.0 | 1670.1 | 1226.6 | 1124.3 | 1697.8 | 2199.6 | 2221.9 | 2181.1 |
| MP | 0.73 | 0.65 | * | 335.3 | 474.0 | 416.0 | 543.5 | 1042.8 | 1478.9 | 2521.2 | 1571.3 | 1725.0 | 1696.0 |
| SG | 0.68 | **0.65** | **0.10** | * | 703.0 | 305.4 | 434.3 | 830.5 | 1362.6 | 2460.0 | 1224.4 | 1380.0 | 1361.1 |
| NS | 0.77 | 0.66 | 0.51 | 0.64 | * | 504.4 | 489.6 | 1499.9 | 1958.6 | 2970.3 | 1838.6 | 2023.9 | 2022.9 |
| RI | 0.82 | 0.91 | 0.95 | 0.96 | 0.93 | * | 164.9 | 1134.7 | 1667.3 | 2745.7 | 1348.7 | 1525.0 | 1521.1 |
| WS | 0.82 | 0.91 | 0.95 | 0.96 | 0.93 | **-0.05** | * | 1250.6 | 1800.3 | 2911.5 | 1388.8 | 1609.2 | 1588.6 |
| B1 | 0.79 | 0.88 | 0.92 | 0.93 | 0.92 | 0.78 | 0.78 | * | 599.6 | 1729.1 | 1029.1 | 1012.1 | 969.3 |
| B2 | 0.79 | 0.86 | 0.91 | 0.93 | 0.89 | 0.97 | 0.98 | 0.95 | * | 1119.2 | 1501.5 | 1387.7 | 1362.0 |
| PP | **0.58** | **0.60** | 0.75 | 0.73 | 0.76 | 0.89 | 0.89 | 0.86 | 0.82 | * | 2516.2 | 2348.4 | 2293.0 |
| CJ | 0.44 | **0.34** | 0.58 | 0.53 | 0.62 | 0.69 | 0.68 | 0.66 | 0.59 | **0.36** | * | 264.5 | 284.9 |
| EJ | 0.73 | **0.75** | 0.87 | 0.89 | 0.86 | 0.95 | 0.95 | 0.92 | 0.91 | 0.69 | **0.27** | * | 37.1 |
| MD | **0.73** | **0.78** | 0.90 | 0.92 | 0.88 | 0.96 | 0.97 | 0.93 | 0.94 | 0.73 | **0.30** | **0.06** | * |

**Note:** Darker shades indicates higher Φ_ST_ values

Numbers in bold indicate non significance at alpha = 0.05 after Bonferroni correction for multiple comparisons

Key: PM- Malayan Peninsula, SG-Singapore, TH-Thailand, VT-Vietnam, NS-North Sumatra, WS-West Sumatra, RI-Riau, CJ-Central Java, EJ-East Java, MD-Madura, PP-the Philippines, B1-southwest Borneo, B2-northwest Borneo.
