# Supplementary material for: Phylogeography of the termite Macrotermes gilvus and insight into ancient dispersal corridors in Pleistocene Southeast Asia
Source: PLoS One. 2017 Nov 29;12(11):e0186690. doi: 10.1371/journal.pone.0186690 (PMC5706666; doi:10.1371/journal.pone.0186690)
Supplement: S8 Table — (DOCX) [file pone.0186690.s008.docx]

**S8 Table. Allelic richness (A_R_), number of alleles (N_A_), number of private alleles (in parenthesis) and its percentages (P_A_) for each locus and population.**

| Locus | Pop | PM | SG | TH | VT | NS | RI | WS | CJ | MD | EJ | B1 | B2 | PP | Total |
| --- | --- | --- | --- | --- | --- | --- | --- | --- | --- | --- | --- | --- | --- | --- | --- |
|  | N | 36 | 10 | 13 | 9 | 21 | 19 | 13 | 14 | 16 | 10 | 27 | 13 | 6 | 207 |
| MG18 | N_A_ | 5 | 4 | 4 | 5 | 3 | 4 | 4 | 6 (1) | 7 | 7 | 4 | 4 | 5 | 10 (1) |
|  | A_R_ | 3.6 | 3.0 | 3.8 | 3.7 | 2.8 | 3.4 | 3.2 | 4.4 | 4.8 | 5.2 | 3.1 | 3.5 | 5.0 | 5.1 |
| MG34 | N_A_ | 4 | 2 | 4 (1) | 2 | 2 | 2 | 2 | 4 | 6 (1) | 4 | 3 | 3 (1) | 2 | 11 (3) |
|  | A_R_ | 2.4 | 1.8 | 3.6 | 1.6 | 1.3 | 2.0 | 1.9 | 2.3 | 4.3 | 2.9 | 2.0 | 3.0 | 2.0 | 4.3 |
| MG2 | N_A_ | 3 | 3 | 3 | 3 | 2 | 1 | 1 | 1 | 3 (1) | 1 | 5 (1) | 2 | 2 | 8 (2) |
|  | A_R_ | 2.2 | 2.0 | 1.8 | 2.8 | 1.6 | 1.0 | 1.0 | 1.0 | 2.1 | 1.0 | 2.9 | 2.0 | 1.8 | 2.3 |
| MG1 | N_A_ | 4 | 2 | 5 (1) | 5 | 2 | 4 | 4 | 5 | 6 | 6 | 1 | 1 | 2 | 9 (1) |
|  | A_R_ | 2.4 | 2.0 | 3.5 | 3.9 | 2.0 | 2.8 | 3.3 | 4.2 | 4.3 | 4.6 | 1.0 | 1.0 | 2.0 | 4.9 |
| MG30 | N_A_ | 5 | 2 | 9 | 6 | 2 | 7 (1) | 6 | 7 | 8 | 6 | 2 | 2 | 7 (2) | 18 (3) |
|  | A_R_ | 2.4 | 2.0 | 6.1 | 5.2 | 1.5 | 4.9 | 4.7 | 4.2 | 4.5 | 4.6 | 1.7 | 2.0 | 6.3 | 5.8 |
| MG37 | N_A_ | 8 (1) | 4 | 10 | 7 | 3 | 6 | 6 | 7 | 8 | 6 | 5 (3) | 5 (1) | 6 | 21 (5) |
|  | A_R_ | 3.8 | 3.5 | 6.2 | 5.7 | 2.3 | 4.1 | 4.3 | 4.2 | 4.2 | 4.6 | 3.8 | 3.7 | 5.6 | 7.0 |
| MG3 | N_A_ | 1 | 1 | 5 | 3 | 1 | 2 | 2 | 5 | 6 (2) | 4 | 4 | 4 | 3 | 8 (2) |
|  | A_R_ | 1.0 | 1.0 | 3.9 | 2.8 | 1.0 | 1.9 | 2.0 | 3.7 | 4.9 | 3.7 | 2.4 | 3.0 | 3.0 | 3.9 |
| MG7 | N_A_ | 2 | 1 | 9 (4) | 3 | 3 | 5 | 4 | 2 | 2 | 2 | 2 | 2 | 3 | 11 (4) |
|  | A_R_ | 1.7 | 1.0 | 5.8 | 2.6 | 2.5 | 4.2 | 3.0 | 1.6 | 2.0 | 1.9 | 2.0 | 2.0 | 3.0 | 4.1 |
| MG5 | N_A_ | 11 (1) | 4 | 9 | 10 | 3 | 4 | 3 | 5 | 10 (2) | 7 | 3 | 4 | 4 | 16 (3) |
|  | A_R_ | 5.9 | 3.5 | 6.2 | 6.9 | 2.9 | 3.1 | 2.6 | 4.1 | 6.5 | 5.1 | 2.3 | 3.0 | 3.7 | 6.1 |
| MG6 | N_A_ | 3 (1) | 2 | 6 (1) | 4 | 3 | 6 | 4 | 6 | 6 | 6 | 1 | 3 (2) | 3 | 12 (4) |
|  | A_R_ | 2.1 | 2.0 | 3.9 | 3.5 | 2.3 | 3.6 | 3.3 | 4.5 | 4.2 | 4.6 | 1.0 | 2.4 | 3.0 | 5.1 |
| MG8 | N_A_ | 4 | 3 | 5 (1) | 9 (1) | 2 | 6 | 4 | 5 | 9 (1) | 5 | 5 | 3 | 5 | 13 (3) |
|  | A_R_ | 2.6 | 2.5 | 3.8 | 6.6 | 1.9 | 4.0 | 3.4 | 3.5 | 5.0 | 4.2 | 3.0 | 2.4 | 4.5 | 4.6 |
| MG11 | N_A_ | 6 (1) | 3 | 4 | 4 | 6 (2) | 7 | 4 | 5 | 5 | 5 | 3 | 2 | 5 | 11 (3) |
|  | A_R_ | 4.0 | 3.0 | 3.7 | 3.9 | 3.8 | 4.5 | 3.8 | 3.6 | 3.9 | 4.0 | 2.6 | 1.8 | 4.7 | 5.2 |
| MG9 | N_A_ | 6 | 4 | 7 | 6 | 3 | 2 | 2 | 2 | 2 | 1 | 1 | 2 | 5 (1) | 11 (1) |
|  | A_R_ | 3.8 | 2.8 | 5.5 | 4.7 | 2.2 | 2.0 | 2.0 | 1.6 | 1.3 | 1.0 | 1.0 | 2.0 | 5.0 | 4.8 |
| MG36 | N_A_ | 3 (1) | 2 | 5 | 1 | 2 | 2 | 2 | 3 | 4 | 1 | 3 | 2 | 3 | 8 (1) |
|  | A_R_ | 2.3 | 2.0 | 4.1 | 1.0 | 2.0 | 2.0 | 2.0 | 2.4 | 3.0 | 4.2 | 1.9 | 2.0 | 3.0 | 4.1 |
| MG33 | N_A_ | 3 | 3 | 9 (4) | 4 | 3 | 3 | 2 | 6 | 5 | 7 (2) | 3 | 2 | 1 | 13 (6) |
|  | A_R_ | 3.0 | 2.5 | 6.4 | 3.4 | 2.4 | 2.6 | 1.6 | 4.2 | 4.1 | 4.9 | 1.7 | 2.0 | 1.0 | 4.7 |
| Average N_A_ | | 4.5 | 2.7 | 6.3 | 4.8 | 2.7 | 4.1 | 3.3 | 4.6 | 5.8 | 4.8 | 3.0 | 2.7 | 3.7 | 12.0 |
| Total N_A_ | | 68 (5) | 40 (0) | 94 (12) | 72 (1) | 40 (2) | 61 (1) | 50 (0) | 69 (1) | 87 (7) | 72 (2) | 45 (4) | 41 (4) | 56 (3) | 180 (42) |
| Average A_R_ |  | 2.88 | 2.31 | 4.55 | 3.89 | 2.17 | 3.07 | 2.81 | 3.30 | 3.94 | 3.77 | 2.16 | 2.39 | 3.57 | 4.80 |
| P_A_ (%) |  | 7.4 | 0.0 | 12.8 | 1.4 | 5.0 | 1.6 | 0.0 | 1.5 | 8.1 | 2.8 | 8.9 | 9.8 | 5.4 | 23.3 |

Key: PM- Malay Peninsula, SG-Singapore, TH-Thailand, VT-Vietnam, NS-North Sumatra, WS-West Sumatra, RI-Riau, CJ-Central Java, EJ-East Java, MD-Madura, PP-the Philippines, B1-southwest Borneo, B2-northwest Borneo
