# Supplementary material for: Phylogeography of the termite Macrotermes gilvus and insight into ancient dispersal corridors in Pleistocene Southeast Asia
Source: PLoS One. 2017 Nov 29;12(11):e0186690. doi: 10.1371/journal.pone.0186690 (PMC5706666; doi:10.1371/journal.pone.0186690)
Supplement: S9 Table — (DOCX) [file pone.0186690.s009.docx]

**S9 Table. Pairwise R_ST_ (sum of squared sized differences – lower diagonal) and F_ST_ (number of different alleles – upper diagonal) generated from Arlequin 2.0.**

|  | Mainland Southeast Asia | | | | Sumatra | | | Borneo | | Philippines | Java | | |
| --- | --- | --- | --- | --- | --- | --- | --- | --- | --- | --- | --- | --- | --- |
|  | TH | VT | PM | SG | NS | RI | WS | B1 | B2 | PP | CJ | EJ | MD |
| TH | * | 0.18 | 0.27 | 0.28 | 0.34 | 0.26 | 0.26 | 0.41 | 0.29 | 0.18 | 0.23 | 0.20 | 0.18 |
| VT | 0.31 | * | 0.23 | 0.27 | 0.34 | 0.27 | 0.30 | 0.46 | 0.35 | 0.25 | 0.28 | 0.25 | 0.25 |
| PM | 0.42 | 0.31 | * | 0.09 | 0.13 | 0.38 | 0.38 | 0.50 | 0.42 | 0.34 | 0.37 | 0.35 | 0.32 |
| SG | 0.36 | 0.30 | **0.06** | * | 0.25 | 0.41 | 0.43 | 0.56 | 0.48 | 0.38 | 0.42 | 0.40 | 0.36 |
| NS | 0.52 | 0.44 | **0.05** | 0.21 | * | 0.42 | 0.43 | 0.58 | 0.52 | 0.44 | 0.46 | 0.45 | 0.40 |
| RI | 0.62 | 0.57 | 0.73 | 0.77 | 0.79 | * | **0.04** | 0.42 | 0.40 | 0.33 | 0.27 | 0.25 | 0.28 |
| WS | 0.64 | 0.61 | 0.76 | 0.80 | 0.82 | **0.04** | * | 0.48 | 0.43 | 0.36 | 0.32 | 0.29 | 0.30 |
| B1 | 0.73 | 0.73 | 0.76 | 0.77 | 0.81 | 0.85 | 0.86 | * | 0.46 | 0.35 | 0.30 | 0.35 | 0.30 |
| B2 | 0.65 | 0.65 | 0.73 | 0.61 | 0.81 | 0.83 | 0.83 | 0.43 | * | 0.26 | 0.35 | 0.34 | 0.29 |
| PP | 0.32 | **0.31** | 0.52 | 0.61 | 0.71 | 0.63 | 0.64 | 0.74 | 0.61 | * | 0.24 | 0.22 | 0.19 |
| CJ | 0.37 | 0.41 | 0.44 | 0.49 | 0.56 | 0.67 | 0.70 | 0.64 | 0.55 | 0.42 | * | **0.05** | 0.09 |
| EJ | 0.46 | 0.48 | 0.52 | 0.59 | 0.65 | 0.72 | 0.75 | 0.71 | 0.61 | 0.51 | **0.07** | * | **0.04** |
| MD | 0.37 | 0.40 | 0.45 | 0.46 | 0.52 | 0.65 | 0.67 | 0.63 | 0.51 | 0.34 | **0.05** | **0.02** | * |

**Note:** Darker shades indicates higher R_ST_/F_ST ­_values

Numbers in bold indicate non significance at alpha = 0.05 after Bonferroni correction for multiple comparisons

Key: PM- Malayan Peninsula, SG-Singapore, TH-Thailand, VT-Vietnam, NS-North Sumatra, WS-West Sumatra, RI-Riau, CJ-Central Java, EJ-East Java, MD-Madura, B1- BorneoI, B2-BorneoII, PP-the Philippines
