# Supplementary material for: Phylogeography of the termite Macrotermes gilvus and insight into ancient dispersal corridors in Pleistocene Southeast Asia
Source: PLoS One. 2017 Nov 29;12(11):e0186690. doi: 10.1371/journal.pone.0186690 (PMC5706666; doi:10.1371/journal.pone.0186690)
Supplement: S1 Fig — (DOCX) [file pone.0186690.s010.docx]

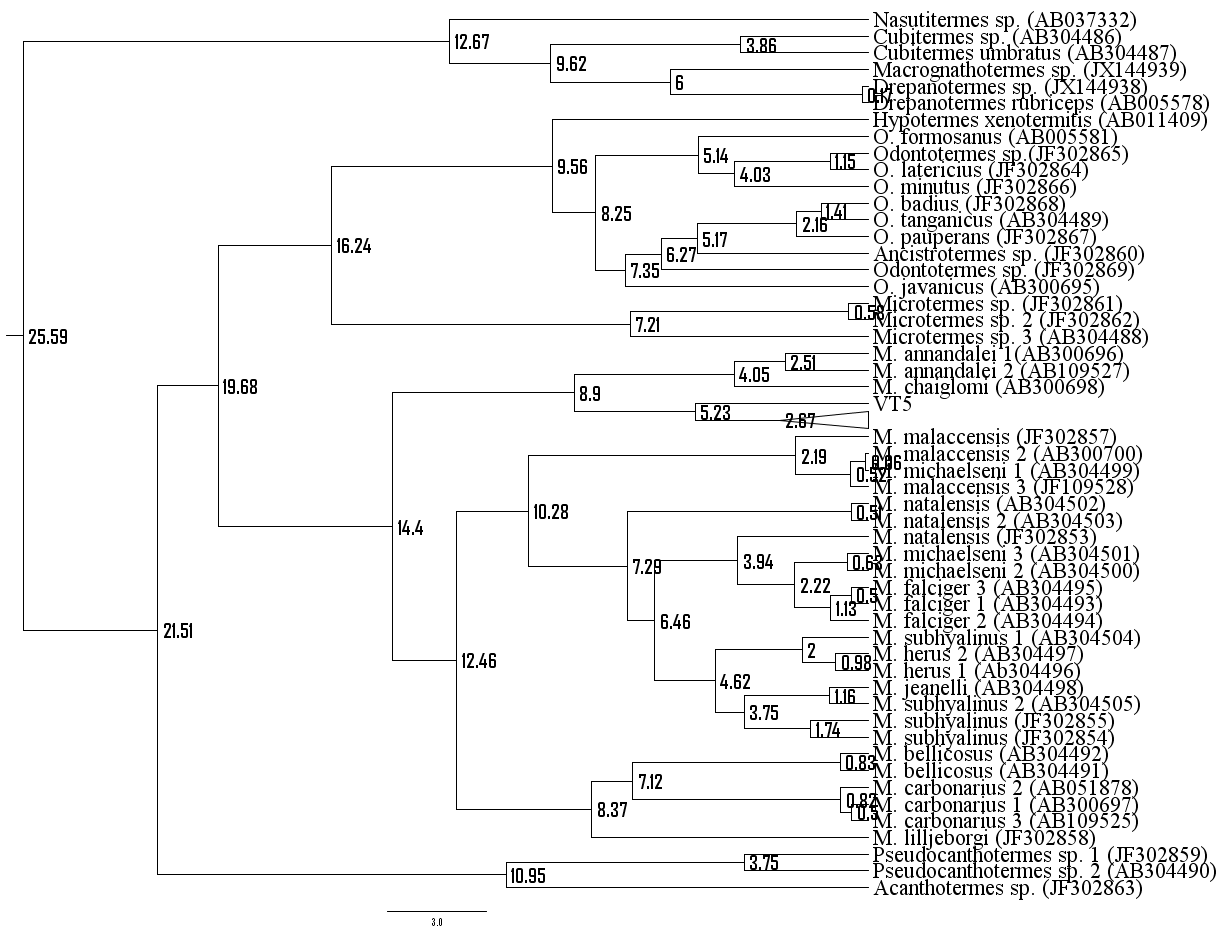


**(a)**

In group (M. gilvus)

**S1 Fig. Divergence dating of the fungus-growing termites based on COII gene.**

**(a)** Two fossil calibrations were used as indicated by the black triangles. The *Odontotermes* node was constrained to a minimum age of 7 Ma following a lognormal distribution (lognormal mean = 1.9, SD = 1.5, zero offset = 7) based on dating of the fossilized fungus comb [86], and a second constraint node with a minimum age of 3.4 Ma (lognormal mean = 1.2, SD = 1, zero offset = 3.4) was also used; it corresponds to the age of the ancestors of the sister species *M. jeanelli* and *M. subhyalinus* based on the fossilized mound described by Darlington [87]. The phylogeny corresponds to the consensus tree from two independent BEAST [39] searches for 100 million generations, each incorporating site-specific substitution models with a tree prior set to “Speciation: Birth-Death process”. Convergence of runs for adequate posterior estimates of divergence times and substitution model parameters was assessed by ensuring the estimated sample size (ESS) values were greater than 200. Internal nodes are labeled with age estimates in unit million years ago. Abbreviations are O, *Odontotermes* and M, *Macrotermes*. The ingroup, *Macrotermes gilvus* clade was collapsed for clarity of overall phylogenetic relationship – attention is called for haplotype VT5 that is placed as outgroup to the *M. gilvus* clade. Outgroup taxa based on Genbank data were chosen from the more distant higher termite groups that are not fungus-growing (*Nasutitermes* sp., *Cubitermes* spp., *Macrognathotermes* sp., and *Drepanotermes* spp.).


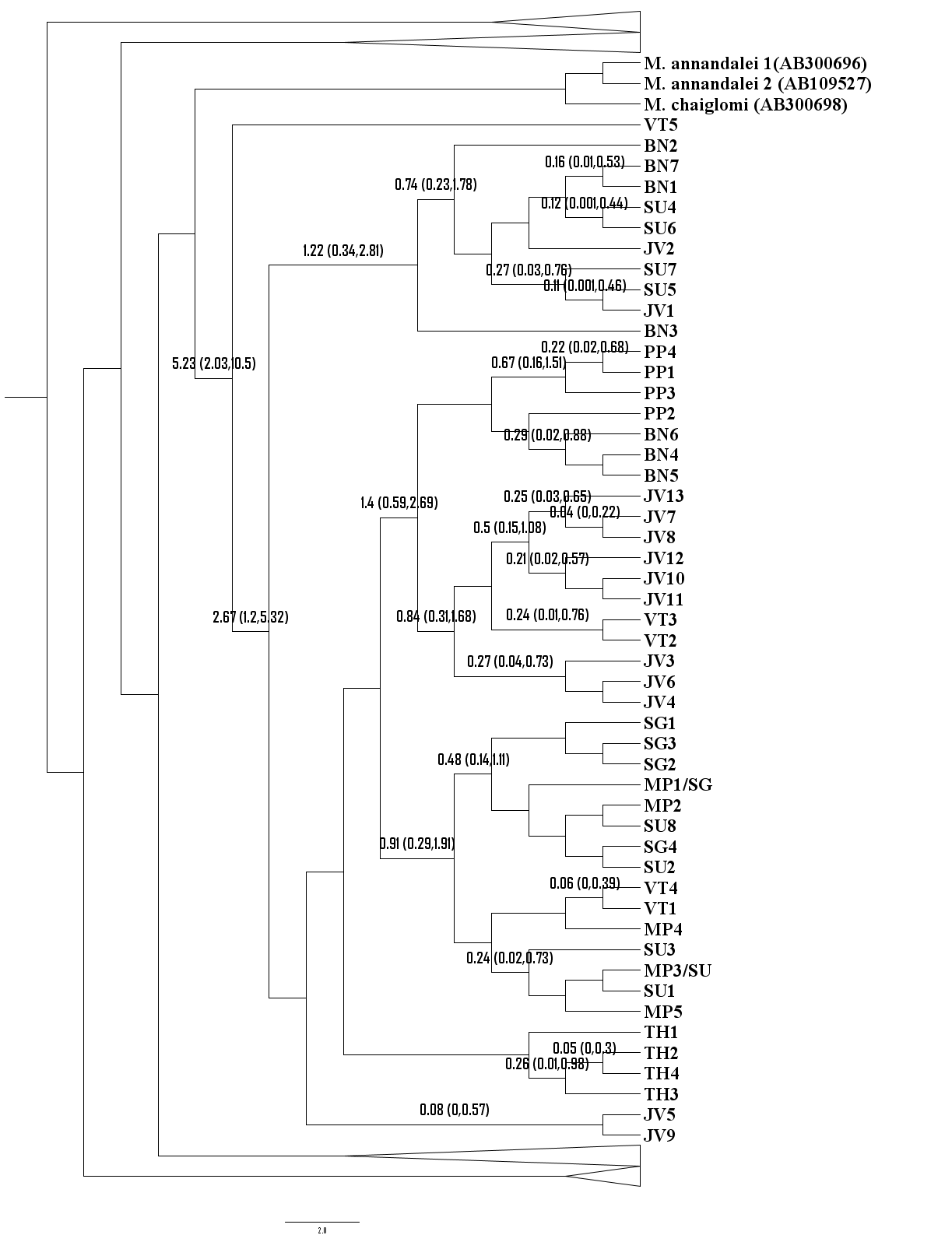


**(b)**

S1 Fig: **(b)** Divergence times of ingroup taxa, *Macrotermes gilvus* (95 % highest posterior densities are shown in bracket). Haplotype abbreviations are referred to S1 Table. Only nodes with posterior support of > 70 % are indicated.

References:

39. Drummond AJ, Rambaut A. BEAST: Bayesian evolutionary analysis by sampling trees. BMC Evol Biol. 2007; 7: 214.

86. Duringer P, Schuster M, Genise JF, Mackaye HT, Vignaud P, Brunet M. New termite trace fossils: galleries, nests and fungus combs from the Chad basin of Africa (Upper Miocene-Lower Pliocene). Palaeogeogr Palaeoclimatol, Palaeoecol. 2007; 251: 323–353.

87. Darlington J. Distinctive fossilized termite nests at Laetoli, Tanzania. Insect Soc. 2005; 52: 408–409.
