# Supplementary material for: Phylogeography of the termite Macrotermes gilvus and insight into ancient dispersal corridors in Pleistocene Southeast Asia
Source: PLoS One. 2017 Nov 29;12(11):e0186690. doi: 10.1371/journal.pone.0186690 (PMC5706666; doi:10.1371/journal.pone.0186690)
Supplement: S1 Appendix — (DOCX) [file pone.0186690.s011.docx]

**S1 Appendix. PCR conditions and primers used for the mtDNA sequencing.**

The following primers were used: (1) COII gene primer pair: Atleu (5’-ATGGCAGATTAGTGCAATGG-3’) and Btlys (5’-GTTTAAGAGAC-CAGTACTTG-3’) [81–82], which targeted a ~681 bp fragment; (2) 16s rRNA gene primer pair: LRJ-13017 (5’-TTACGCTGTTATCCCTAA-3’) and LRN-13398 (5’-CGCCTGTTTATCAAAAACAT-3’) [82], which targeted a ~426 bp fragment. Each PCR was carried in a 25 µl reaction volume on a thermal cycler (PTC 200, MJ Research Inc., Waltham, MA) with amplification conditions as follow: (1) COII gene: an initial denaturing at 94 ºC for 3 min, followed by 35 cycles of denaturing at 94 ºC for 30 sec, annealing at 54.2 ºC for 30 sec, and extension at 65 ºC for 3 min (2) 16s rRNA gene: an initial denaturing at 94 ºC for 5 min, followed by 35 cycles of denaturing at 94 ºC for 1 min, annealing at 46 ºC for 1 min, and extension at 70 ºC for 2 min. Purified PCR were sent to First BASE Laboratories (Selangor, Malaysia) for bidirectional sequencing on an ABI 3730 automated sequence analyzer (Applied Biosystems, Foster City, CA).

Multiple DNA sequences were initially aligned independently for both genes using MEGA 4.0 [83] under default settings. Subsequently, sequence alignments from both genes were concatenated as the incongruence length difference (ILD) test implemented in PAUP* 4.10b resulted in homogeneity between the two genes (P > 0.05). This finding suggested congruency in evolutionary history and selection processes, as the genes are structurally linked in the mitochondrial genome. Hence, we treated the two gene loci as a single unit mtDNA marker in all subsequent analyses unless specified otherwise. Mitochondrial haplotypes for the concatenated dataset were then summarized by using DNASP 5.10 [84].

References

81. Liu H, Beckenbach AT. Evolution of the mitochondrial cytochrome oxidase II gene among 10 orders of insects. Mol Phylogenet Evol. 1992; 41: 41–52.

82. Simon C, Frati F, Beckenback A, Crespi B, Liu H, Flook P. Evolution, weighting, and phylogenetic utility of mitochondrial gene sequences and a compilation of conserved polymerase chain reaction primers. Ann Entomol Soc Am. 1994; 87: 651–701.

83. Tamura K, Dudley J, Nei M, Kumar S. MEGA4: Molecular Evolutionary Genetics Analysis MEGA software version 4.0. Mol Biol Evol. 2007; 10: 1093/ molbev/msm092.

84. Librado P, Rozas J. DNAsp v5: a software for comprehensive analysis of DNA polymorphism data. Bioinformatics. 2009; 25: 1451–1452.
