# Supplementary material for: Phylogeography of the termite Macrotermes gilvus and insight into ancient dispersal corridors in Pleistocene Southeast Asia
Source: PLoS One. 2017 Nov 29;12(11):e0186690. doi: 10.1371/journal.pone.0186690 (PMC5706666; doi:10.1371/journal.pone.0186690)
Supplement: S2 Appendix — (DOCX) [file pone.0186690.s012.docx]

From: Harold Voris

Sent: Thursday, August 17, 2017 9:54 PM

To: Veera Singham A/L K.Genasan

Cc: EDLIC A/L SATHIAMURTHY

Subject: Re: Pleistocene Maps Request

Dear Dr. G. Veera Singham,

Thank you for your email and your interest in our published maps. Yes, you may include Figure 2 from our paper “Sathiamurthy E, Voris HK. Maps of Holocene sea level transgression and submerged lakes on the Sunda shelf published in The Natural History Journal of Chulalongkorn University. 2006; 2: 1–43” in the open-access journal PLOS ONE. This message provides explicit written permission to publish the figure 2 map under a CC BY licence.

Best wishes,

Harold Voris

---------------------------------------------------------------------------------

Visit some of our web sites highlighting our research.

Aquatic Snakes of Southeast Asia at:

http://archive.fieldmuseum.org/aquaticsnakes/index.html

Sea Level Maps of Sundaland during the Pleistocene at:

http://fieldmuseum.org/explore/pleistocene-sea-level-maps

Research and Collections in the Division of Amphibians and Reptiles at:

http://fieldmuseum.org/explore/department/zoology/amphibians-and-reptiles/people

Harold K. Voris, Ph.D.

Curator Emeritus

Science and Education

Field Museum of Natural History

1400 South Lake Shore Drive

Chicago, Illinois 60605

1 312 665 7769

http://fieldmuseum.org/users/harold-k-voris

------------------------------------------------------------

On Wed, Aug 16, 2017 at 2:34 AM, Veera Singham A/L K.Genasan <veerasingham@usm.my> wrote:

Dear Dr. Voris,

I am Veera Singham, currently a lecturer from University Science Malaysia would like to request permission for the open-access journal PLOS ONE to publish the map of Fig.2 from the journal “Sathiamurthy E, Voris HK. Maps of Holocene sea level transgression and submerged lakes on the Sunda shelf. The Natural History Journal of Chulalongkorn University. 2006; 2: 1–43” with modifications under the Creative Commons Attributions Licence (CCAL) CC BY 4.0 (http://creativecommons.org/licences/by/4.0/). Please be aware that this license allows unrestricted use and distribution, even commercially, by third parties. Please reply and provide explicit written permission to publish the figure map as mentioned under a CC BY licence.

I sincerely thank you for your kind consideration in allowing me to use the map for the journal publication.

Please do not hesitate to contact me for further information.

Thank you and best regards,

Veera

Dr. G. Veera Singham

Centre for Chemical Biology,

Universiti Sains Malaysia

Sains@USM, level 1, Block B

10 Persiaran Bukit Jambul,

11900 Bayan Lepas, Pulau Pinang, Malaysia

Contact: +604-653 5534 Fax: +604-653 5514

URL:www.ccb.usm.my

Sent from Mail for Windows 10
